# The Transcriptional Response of *Candida albicans* to Weak Organic Acids, Carbon Source, and *MIG1* Inactivation Unveils a Role for *HGT16* in Mediating the Fungistatic Effect of Acetic Acid

**DOI:** 10.1534/g3.117.300238

**Published:** 2017-09-06

**Authors:** Fabien Cottier, Alrina Shin Min Tan, Marina Yurieva, Webber Liao, Josephine Lum, Michael Poidinger, Francesca Zolezzi, Norman Pavelka

**Affiliations:** Singapore Immunology Network (SIgN), Agency for Science, Technology and Research (A*STAR), Singapore 138648, Singapore

**Keywords:** short-chain fatty acid, transcriptomics, gene expression profiling, RNA-seq, glucose repression, *MIG1*, *Candida albicans*

## Abstract

*Candida albicans* is a resident fungus of the human intestinal microflora. Commonly isolated at low abundance in healthy people, *C. albicans* outcompetes local microbiota during candidiasis episodes. Under normal conditions, members of the human gastrointestinal (GI) microbiota were shown to keep *C. albicans* colonization under control. By releasing weak organic acids (WOAs), bacteria are able to moderate yeast growth. This mechanism displays a synergistic effect *in vitro* with the absence of glucose in medium of culture, which underlines the complex interactions that *C. albicans* faces in its natural environment. Inactivation of the transcriptional regulator *MIG1* in *C. albicans* results in a lack of sensitivity to this synergistic outcome. To decipher *C. albicans* transcriptional responses to glucose, WOAs, and the role of *MIG1*, we performed RNA sequencing (RNA-seq) on four biological replicates exposed to combinations of these three parameters. We were able to characterize the (i) glucose response, (ii) response to acetic and butyric acid, (iii) *MIG1* regulation of *C. albicans*, and (iv) genes responsible for WOA resistance. We identified a group of six genes linked to WOA sensitivity in a glucose-*MIG1*-dependent manner and inactivated one of these genes, the putative glucose transporter *HGT16*, in a SC5314 wild-type background. As expected, the mutant displayed a partial complementation to WOA resistance in the absence of glucose. This result points toward a mechanism of WOA sensitivity in *C. albicans* involving membrane transporters, which could be exploited to control yeast colonization in human body niches.

Considered as a human commensal, *Candida albicans* is frequently isolated from the human GI tract where it coexists harmlessly with its host. However, this yeast can become virulent, particularly when associated with certain pathologies such as immunodepression or antibiotic treatment (Yapar 2014; Pfaller and Diekema 2007). At the core of *C. albicans* virulence is its dimorphic nature, an ability to switch between blastospore and filamentous forms, allowing *C. albicans* to invade tissue and evade immune cells (Saville *et al.* 2003; Krysan *et al.* 2014). This morphological switch of *C. albicans* occurs in response to numerous external stimuli such as temperature, pH, hypercapnia, serum, and mannitol or ammonium concentrations. Cell growth can also be impacted by as many conditions and demonstrates how responsive the yeast is to its environment. In its natural niches (GI tract, vagina, and skin), some of these signals, such as WOAs, are metabolites originating from the local bacterial population. For example, *Lactobacillus*, predominant genus of the vaginal microbiota, produces lactic acid to control *C. albicans* colonization in healthy women via WOA acidification (Osset *et al.* 2001; Kohler *et al.* 2012). On the other hand, acetic and butyric acids are synthesized during anaerobic fermentation by bacteria present in the human GI tract (Mortensen and Clausen 1996), which are able to reduce the growth rate of *C. albicans in vitro* as well as decrease yeast GI colonization in mice (Fan *et al.* 2015). Indeed, addition of 50 mM of acetic acid to the drinking water of mice reduced the number of live *C. albicans* per gram of stool by a factor of 10 in antibiotic-treated mice. We and others have previously confirmed this negative impact of WOAs on *C. albicans* growth *in vitro* (Cottier *et al.* 2015a; Huang *et al.* 2011). We demonstrated that when exposed at concentrations of acetic and butyric acid similar to the ones identified in human stool, the *C. albicans* growth rate was reduced by ∼50%. This triggered upregulation of genes involved in iron transport and an opposite effect on RNA synthesis genes (Cottier *et al.* 2015a). We further identified the transcriptional regulator *MIG1* as a central contributor to WOA resistance. *MIG1* inactivation increased sensitivity to acetic acid by 80% and by 20% for lactic and butyric acid in Yeast extract-Peptone-Dextrose (YPD) media (Cottier *et al.* 2015b). In the model yeast *Saccharomyces cerevisiae*, Mig1p is known as a major regulator involved in glucose repression. In the presence of glucose, this zinc finger protein represses the expression of genes encoding proteins involved in the utilization of nonglucose sugars such as maltose or sucrose. Similarly, in *C. albicans*, inactivation of *MIG1* has been associated with the derepression of gluconeogenic genes like *PCK1* (Murad *et al.* 2001). When *C. albicans* is grown on maltose as a main carbon source instead of glucose, a wild-type strain of *C. albicans* displays an increased sensitivity to WOAs by ≤ 75% for acetic and butyric acid (Cottier *et al.* 2015b). This mimics the phenotypes observed with the *MIG1* deletion mutant.

To identify which genes are involved in *MIG1*-glucose-dependent sensitivity to WOAs in *C. albicans*, we investigated the transcriptomic responses of this yeast to acetic and butyric acid in the presence of glucose or maltose for a wild-type and *MIG1* mutant strain. Using RNA-seq, this multiparametric design allowed us to characterize (i) the glucose response, (ii) the response to acetic and butyric acid, (iii) the *MIG1* regulation of *C. albicans*, and (iv) the *MIG1*-glucose-dependent genes involved in WOA sensitivity. The latter represented a group of six genes whose expression is significantly altered between wild-type and *MIG1* mutant strains; but also between the different sources of carbon in the presence of WOAs. We focused our attention on one of them, *HGT16*, a putative glucose transporter that displayed a strong correlation with cell growth rate. We demonstrated that inactivation of this gene induced a partial recovery of acetic acid resistance in glucose-deprived media. This multifactorial analysis sheds light on *C. albicans* responses to common environmental cues, and will help to investigate the synergistic effect of different signals on *C. albicans* growth. Such an approach can support the development of innovative mechanisms to control *C. albicans* colonization.

## Materials and Methods

### Strains and culture conditions

The wild-type *C. albicans* strain SC5314 (Noble and Johnson 2005) and a *MIG1* mutant (Cottier *et al.* 2015b) were used throughout this study. Yeasts strains were grown in YPD media (1% w/v yeast extract, 2% w/v peptone, and 2% w/v d-glucose, supplemented with 1.5% w/v agar for solid media only) or YPM (identical composition to YPD except d-glucose was replaced by maltose) at 37° in a shaking incubator at 150 rpm. Stock cultures were preserved in 35% glycerol and maintained at −80°. Acetic and butyric acid (from either Merck or Sigma) were added to freshly relaunched overnight cultures, diluted down to an optical density at 600 nm of 0.1.

### RNA-seq

Cells were harvested by centrifugation and the pellet was flash-frozen in liquid nitrogen. RNA extractions and sequencing were performed identically to the previously described protocol (Cottier *et al.* 2015a). Raw and processed sequencing data are available on the Gene Expression Omnibus (GEO) database (http://www.ncbi.nlm.nih.gov/geo/) under accession number GSE99767.

### Data analysis

Reads were processed following the previously described protocol of Cottier *et al.* (2015a) using the *C. albicans* genome annotation (Assembly 21, version s02-m08-r09). Reads Per Kilobase per Million mapped reads (RPKM) were calculated in R (3.1.2). Statistical analysis was performed after addition to all values of the lowest RPKM measurement, then data were log10 transformed. Differentially expressed transcripts were identified in Prism (GraphPad) by a series of *t*-tests assuming unequal variance, followed by the two-stage linear step-up procedure of Benjamini, Krieger, and Yekutieli, with *Q* = 1%. Subsequently, only transcripts displaying an average expression ratio <0.5 (for downregulated transcripts) or >2 (for upregulated transcripts) were retained.

Gene ontology (GO) enrichment analysis was performed with the CGD GO Term Finder (Inglis *et al.* 2012), with *P*-values corresponding to Bonferroni-corrected hypergeometric test *P*-values. All other statistical analyses were performed in Microsoft Excel 2013 or Prism and, unless otherwise indicated in the respective figure legends, we used unpaired *t*-tests assuming unequal variances and error bars corresponded to SEM.

### Data availability

Strains are available upon request. Supplemental Material, File S2 contains additional descriptions of materials and methods, figures, and tables. File S1 contains RPKM values for each gene in all conditions. Gene expression data are available at GEO with the accession number GSE99767.

## Results

### Wild-type and MIG1 mutant sensitivity to WOAs in the presence or absence of glucose

Our previous works (Cottier *et al.* 2015a,b) characterized the transcriptional response of *C. albicans* to WOAs in MRS media pH 5.5, and identified Mig1p as a general transcriptional factor involved in WOA sensitivity in YPD media pH 5.5. The latter effect was dependent on the carbon source, as yeast were more sensitive to WOAs when glucose was replaced by maltose in YPD media. We reproduced those results in YPD and YPM media pH 5.5 supplemented with and without acetic or butyric acid at their respective inhibitory concentration of 50% (IC_50_) for both wild-type (SC5314) and the *mig1*Δ strain. As expected, a growth rate reduction of ∼50% was observed for the wild-type cells in the presence of acetic or butyric acid compared to YPD alone (Figure S1 in File S2). This increased sensitivity to WOAs between the two strains was not observed when cells were grown in YPM media. These results are in agreement with our previous observation (Cottier *et al.* 2015b). The *MIG1* mutant displayed a significantly higher sensitivity to both acetic and butyric acid than the control strain under the same conditions, respectively, 75 and 20%. We also confirmed that the SC5314 strain displayed a significantly higher sensitivity to acetic and butyric acid when grown in YPM compared to YPD media. As these results were in concordance with our previously published data (Cottier *et al.* 2015b), we collected cells at the end of the 6 hr growth assay and extracted RNA from the 12 conditions tested.

### Transcriptional responses of C. albicans

**Figure 1:**
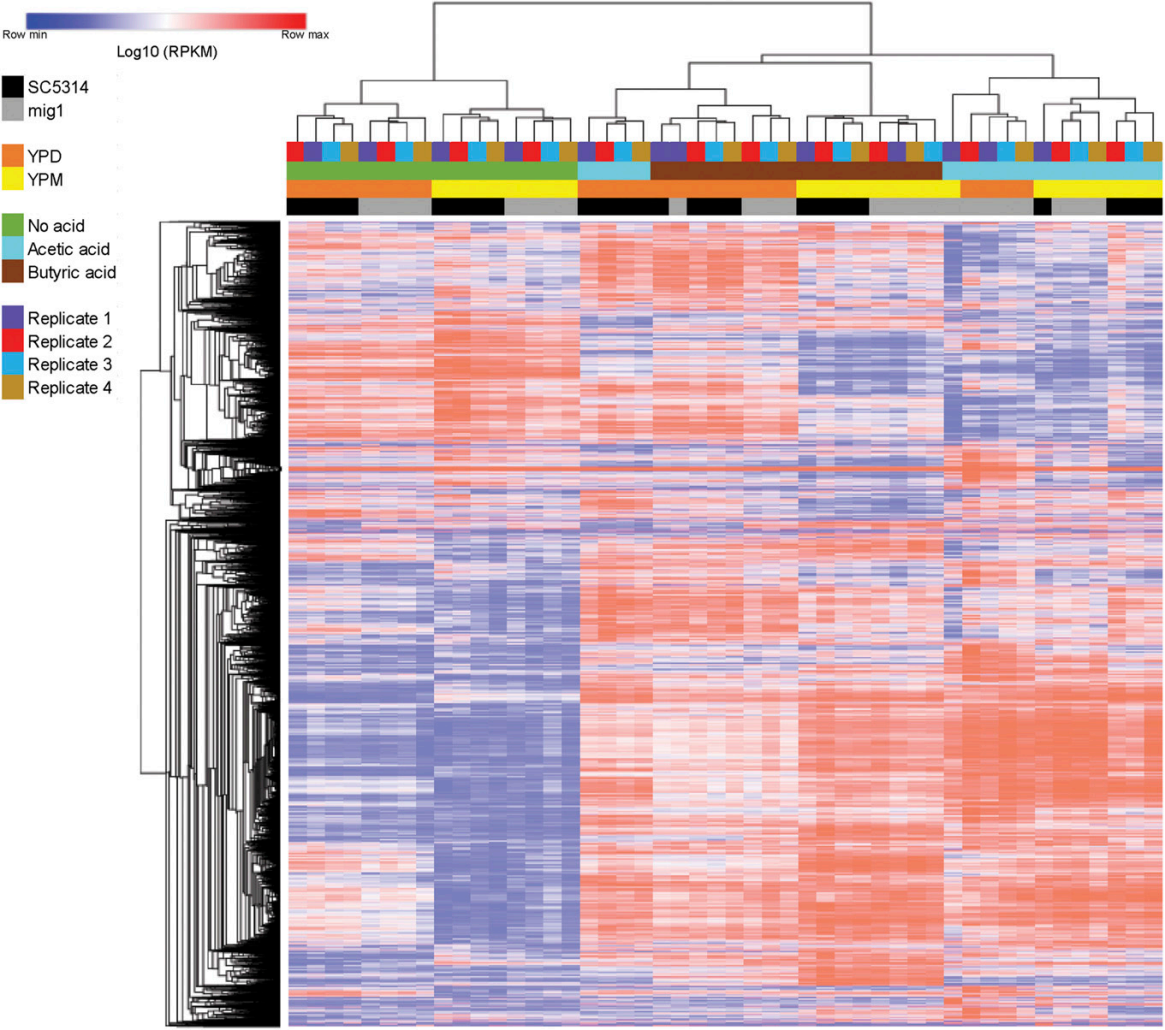
Global transcriptional response of *C. albicans* to carbon source, WOAs, and *MIG1* inactivation. The figure displays a two-way hierarchical clustering of 6218 transcripts (rows) and 48 samples (columns). Log10-transformed RPKM expression values were converted to z-scores, with red indicating expression levels above and blue symbolizing expression levels below the mean expression level of each gene across the samples. Distance metric = 1 − Pearson correlation. RPKM, reads per kilobase per million mapped reads; WOAs, weak organic acids; YPD, yeast extract-peptone-dextrose media; YPM, media with identical composition to YPD except d-glucose replaced by maltose.

We prepared cDNA libraries from the total RNA extracted for all 48 independent samples (12 conditions and four biological replicates); sequencing was performed on an Illumina HiSeq 2000 platform using 2 × 50 bp reads. We obtained an average of 1.4 × 10^7^ (± 2.5 × 10^6^) reads per sample, which were mapped to 6453 transcripts, including both open reading frames and noncoding RNAs. After processing of the reads, we reported gene expression as RPKM values. Hierarchical clustering (Figure 1) of the 48 samples displayed a clear separation principally to WOA treatment, followed by carbon source (YPD or YPM), and finally strains. Replicates were closely clustered together (except for four samples of replicate one) and indicative of a good reproducibility. To investigate the transcriptional responses to (i) glucose (YPD *vs.* YPM), (ii) *MIG1* (SC5314 *vs.* *mig1*Δ in YPD media), (iii), acetic and butyric acid in YPD, and (iv) WOAs and carbon source simultaneously, we performed pair-by-pair comparisons. We obtained lists of genes significantly regulated between two conditions, and focused our attention on genes with at least a twofold regulation. For each comparison, we performed global functional analysis of these gene lists to gain biological insights into the transcriptional responses. We found statistically significantly enriched GO Biological Process terms among the transcripts significantly up- or downregulated.

### Glucose response

**Figure 2:**
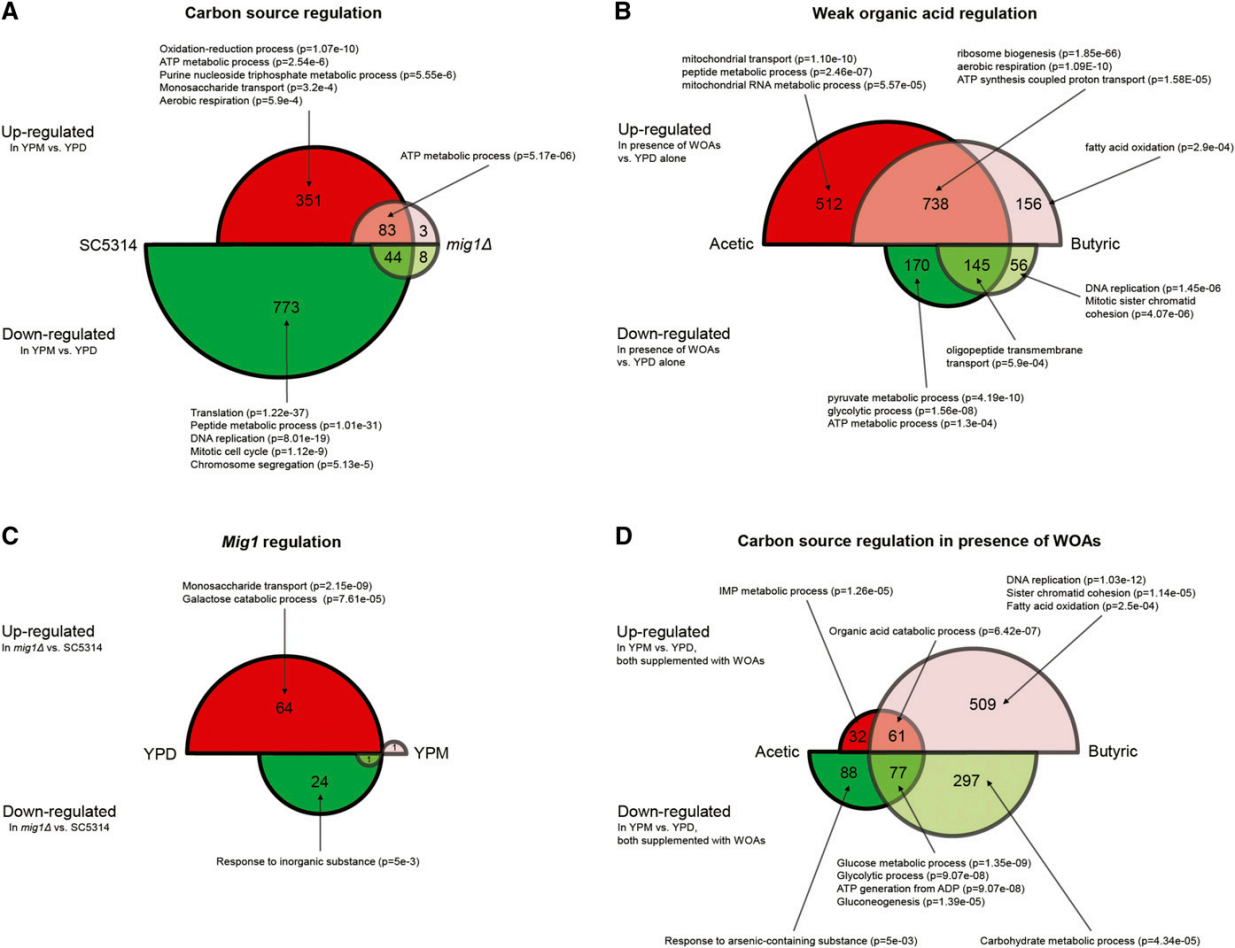
Significantly regulated genes in response to different growth conditions. (A) Genes significantly regulated in response to glucose and maltose. Up- and downregulated genes obtained by comparison of transcriptional profiles between YPM over YPD media for SC3514 and *mig1*Δ strains. (B) Genes significantly regulated in response to WOAs. Up- and downregulated genes obtained by comparison of transcriptional profiles between YPD supplemented with acetic or butyric acid over YPD media for SC3514 strain. Not represented: one gene (*orf19.3684*) displayed opposite regulation between acetic acid (downregulation) and butyric acid (upregulation), and one with the opposite trend (*orf19.7042*). (C) Genes significantly regulated in *MIG1* mutant. Up- and downregulated genes obtained by comparison of transcriptional profiles between *mig1*Δ over the SC3514 strain in YPD and YPM media. (D) Genes significantly regulated in response to carbon source in presence of WOAs. Up- and downregulated genes obtained by comparison of transcriptional profiles between YPM supplemented with acetic or butyric acid over YPD media supplemented with identical acids for the SC3514 strain. Not represented: nine genes displayed opposite regulation between acetic acid (downregulation) and butyric acid (upregulation). WOAs, weak organic acids; YPD, yeast extract-peptone-dextrose media; YPM, media with identical composition to YPD except d-glucose replaced by maltose.

*C. albicans* is frequently grown in the laboratory in rich media, like YPD, which contains 2% glucose, but this concentration can be as low as 0.05% in some body niches (*e.g.*, the bloodstream) (Rodaki *et al.* 2009) or absent, and also subdued by more complex sugars like in the GI tract. To study the impact of an alternative carbon source on *C. albicans*, we selected a close comparative: maltose, a disaccharide formed by two units of glucose, which is produced in the gut during starch breakdown and alters the wild-type *C. albicans* strain growth rate by only <10% (Figure S1 in File S2). Additionally, maltose was shown in *S. cerevisiae* to alleviate glucose repression almost as effectively as *MIG1* inactivation (Klein *et al.* 1996). We first compared the transcript profile between YPM and YPD media for the SC5314 strain; we identified 434 genes upregulated and 817 downregulated in the presence of maltose (Figure 2A). This result was expected as Mig1p is a central regulator of glucose repression (Zaragoza *et al.* 2000). These numbers were, respectively, 86 and 52 in a *MIG1* mutant background, demonstrating that only 12% of the genes regulated by glucose were *mig1*-independent. Downregulated genes were significantly enriched in processes related to translation and the cell cycle, as anticipated from the decrease in *C. albicans* growth rate when exposed to maltose and in agreement with previous data obtained in *S. cerevisiae* (Ashe *et al.* 2000). On the other hand, among the upregulated genes, ATP metabolism and monosaccharide transport were activated. Indeed, 11 transporter genes displayed a higher expression level (*GAL1*, *HGT13*, *HGT17*, *HGT19*, *HGT6*, *HGT7*, *HGT8*, *HXK2*, *HXT5*, *RGT1*, and *orf19.4090*). Maltose transporter (*MAL31*), maltase (*MAL32*), and α-glucosidase (*MAL2*) were all upregulated by a factor of at least 17. This regulation is indicative of an adaptation of *C. albicans* to an alternative carbon source.

### Acetic and butyric acid responses

We formerly described the transcriptional response of *C. albicans* to lactic, acetic, butyric, and propionic acid in MRS media, a glucose-rich media (2%), at pH 5.5 (Cottier *et al.* 2015a). We now performed a similar analysis in YPD media with acetic and butyric acid at IC_50_ with the identical SC5314 strain. Compared to the control condition (YPD), addition of acetic acid to the media induced upregulation of 1251 genes and downregulation of 316, while these numbers were, respectively, 895 and 202 for butyric acid (Figure 2B). Of these genes, 50% shared a similar regulation with both acids, and displayed a significant enrichment in genes related to ATP synthesis and mitochondrial respiration. Despite the reduction in growth induced by the presence of acids, *C. albicans* cells increased gene expression related to the production of energy. In our previous study, these processes were similarly enriched in upregulated genes at early time points. In this study, we also report a significant enrichment in upregulated genes for the processes of ribosome biogenesis and RNA processing in YPD media, but these were observed to be strongly downregulated in MRS media (Cottier *et al.* 2015a). To elucidate this discrepancy, we compared the results from this and our previous study. The transcriptional profiles of SC5314 in MRS and YPD media with or without acetic or butyric acid were analyzed, and we observed major differences between the two media (Figure S2 in File S2). Indeed, close to 61% of *C. albicans* genes were significantly regulated between MRS and YPD alone at pH 5.5. *C. albicans* gene expression was then strongly affected by the medium of culture, and by extension alters the transcriptional responses to WOAs. This observation might explain why, contrary to our previous dataset in MRS media (Cottier *et al.* 2015a), exposure to WOAs in YPD media did not revealed significant expression of genes encoding iron transporters. Under those conditions, only 52 genes were similarly regulated in response to acetic acid in addition to the media of culture (MRS or YPD); and in agreement with our previous analysis, genes involved in arginine biosynthesis (*ARG1*, *ARG3*, *ARG5*,*6*, *CPA1*, and *CPA2*) were upregulated in both media.

### Mig1 response

We previously demonstrated the central role of Mig1p in WOA sensitivity, as the *MIG1* mutant showed a decreased growth rate in response to lactic, acetic, propionic, and butyric acids when compared to the SC5314 control strain (Cottier *et al.* 2015b). To investigate which Mig1p targets were involved in this phenotype, we first characterized the transcriptional profile of the *MIG1* mutant against the SC5314 control strain in YPD media. Inactivation of the transcription factor lead to the upregulation of 64 genes and downregulation of 25 (Figure 2C). As Mig1p has been described by others to be a gene repressor (Zaragoza *et al.* 2000; Murad *et al.* 2001), it was expected that a higher number of genes would be upregulated in the mutant strain. This group was enriched in genes involved in monosaccharide transporter (*GAL1*, *HGT1*, *HGT16*, *HGT19*, *HGT2*, *HGT6*, *HGT7*, *HXK2*, and *HXT5*) and alternative carbon source utilization like galactose. Similarly to *S. cerevisiae*, Mig1p in *C. albicans* is responsible for the repression of the hexose membrane transporter in the presence of glucose (Ozcan and Johnston 1999). In *S. cerevisiae*, Mig1p is known to be phosphorylated by Snf1p in the absence of glucose, prompting a displacement of Mig1p from the nucleus to the cytoplasm and relieving its repression of genes (Treitel *et al.* 1998; De Vit *et al.* 1997). Thus, as in the absence of glucose where Mig1p is localized outside the nucleus, a *mig1*Δ strain should provide a similar profile of expression as a wild-type strain in YPM media. Indeed, only two genes were observed as differentially expressed between the two strains in the presence of maltose. This result is reproduced phenotypically as no significant difference in growth rate is observed between the SC5314 and *mig1*Δ strains in any conditions involving YPM media (Figure S1 in File S2).

### Genes involved in MIG1-glucose-dependent WOA resistance

We observed that inactivation of *MIG1* induced a higher sensitivity to WOAs in YPD media compared to the control strain, and similarly that wild-type strain SC5314 displayed a significantly lower growth rate in the presence of WOAs while grown in YPM *vs.* YPD media (Figure S1 in File S2). As the latter effect is absent in a *MIG1* mutant, we hypothesized that Mig1p targets involved in WOA sensitivity were also regulated between YPD and YPM supplemented with WOAs in the SC5314 background. We performed pairwise comparisons between the transcript profiles of the wild-type strain grown in YPD with acetic acid *vs.* YPM with acetic acid, and identically with butyric acid. Ninety-three genes were upregulated in the presence of acetic acid and 174 downregulated. These numbers were 579 and 374, respectively, for butyric acid (Figure 2D). As expected, in the presence of maltose, processes involved in glucose metabolism were downregulated with both acids. A total of 138 genes were similarly regulated by both conditions.

**Figure 3:**
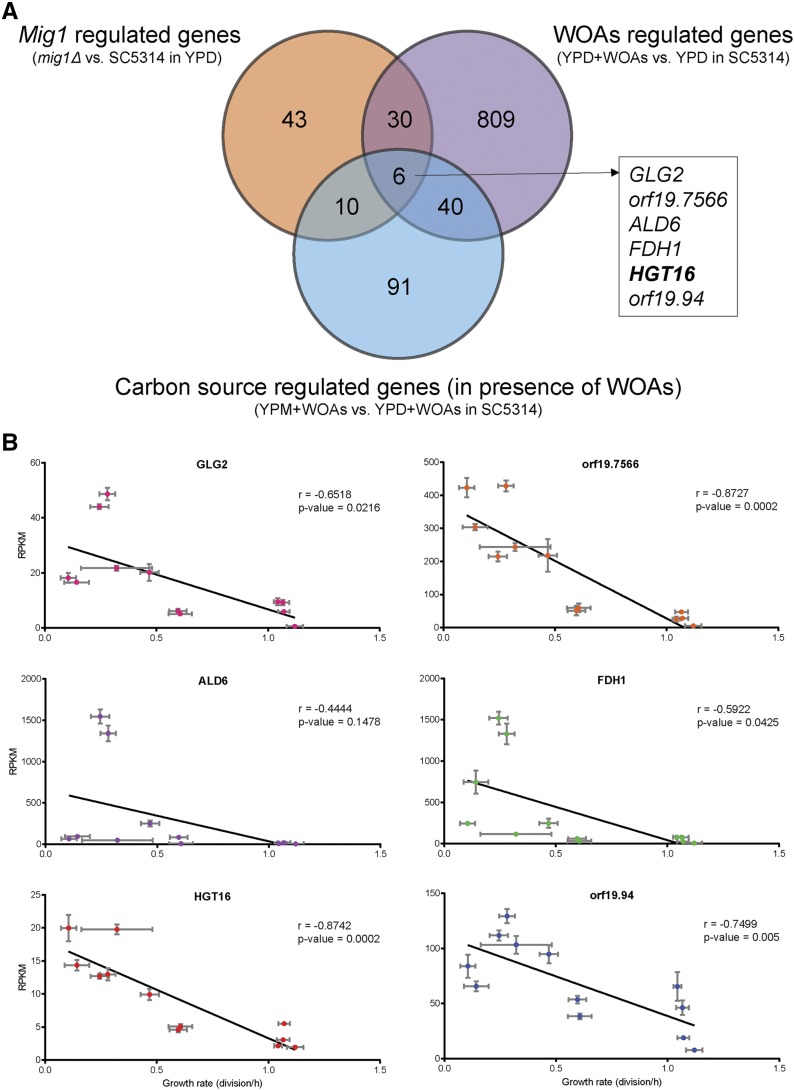
Identification of *MIG1*-glucose-dependent genes involved in WOA sensitivity. (A) Comparison of genes found significantly regulated by *MIG1* (89), presence of WOAs in YPD media (885), and carbon source in the presence of WOAs (147). A group of six genes was identified as commonly present in all three lists. (B) Mean of the RPKM values plotted according to mean growth rates of the four replicates per condition for *GLG2*, *orf19.7566*, *ALD6*, *FDH1*, *HGT16*, and *orf19*.*94*. Solid black lines represent linear regression. Gray error bars represent SEM. RPKM, reads per kilobase per million mapped reads; WOAs, weak organic acids; YPD, yeast extract-peptone-dextrose media; YPM, media with identical composition to YPD except d-glucose replaced by maltose.

Our final objective was the identification of genes involved in WOA sensitivity, where the impact on the phenotype is further increased by the inactivation of *MIG1* or in the absence of glucose. Using our multifactorial analysis, we can now compare the list of genes significantly regulated by (i) WOAs, (ii) *MIG1*, and (iii) carbon source during WOAs exposure. As previously stated, *C. albicans* exposure to acetic or butyric acid led to the common regulation of 885 genes, while Mig1p was responsible for the regulation of 89 genes. Finally, we identified a set of 147 genes in which expression is altered between YPD and YPM media in the presence of WOAs. By comparing these three lists (Figure 3A), we noticed an intersect for only six genes (*GLG2*, *orf19.7566*, *ALD6*, *FDH1*, *HGT16*, and *orf19.94*). All six genes were upregulated under conditions where *C. albicans* sensitivity to WOAs was enhanced, implicating that the functions of these genes are associated with increasing sensitivity to WOAs rather than inhibition of a resistance mechanism. We performed a correlation analysis between profile of expression of these six genes and the growth rate reported for each condition (Figure 3B). As expected, we obtained negative Pearson coefficient factors between the two sets of data, and all correlations were significant (*P* < 0.05), except for *ALD6*. Interestingly, the strongest coefficient factors were obtained for *HGT16* and *orf19.7566*, at −0.874 and −0.872, respectively. Both genes encode putative transmembrane proteins with 11 transmembrane domains. Due to their transmembrane localization, these proteins might have an impact on plasma membrane integrity and molecular diffusion.

### HGT16 inactivation restores acetic acid resistance

**Figure 4:**
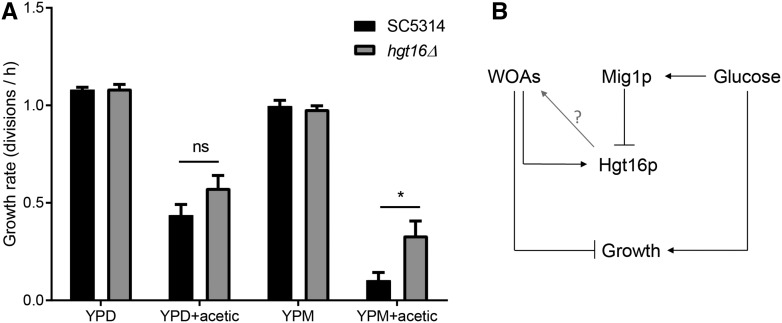
*HGT16* inactivation increases acetic acid resistance. (A) Quantitative growth assays of control (SC5314) and *hgt16*Δ strain performed in YPD or YPM supplemented with acetic acid, at IC_50_ value and adjusted to pH 5.5. * *P* < 0.05. (B) Proposed model where glucose via Mig1p inactivates *HGT16* expression, which is itself stimulated by the presence of WOAs in growth medium. Production of Hgt16p could increase WOA import, which leads to a reduction in *C. albicans* growth rate. WOAs, weak organic acids; YPD, yeast extract-peptone-dextrose media; YPM, media with identical composition to YPD except d-glucose replaced by maltose.

Following the identification of putative targets involved in *MIG1*-glucose-dependent WOA sensitivity, we hypothesized that cell membrane transporters could actively or passively transport WOAs, potentially enhancing their impact on cellular processes. Therefore, as a proof of concept, we decided to inactivate the putative glucose transporter of the major facilitator superfamily: *HGT16* (*orf19.6141*). Both alleles of *HGT16* were inactivated using the *SAT1*-flipping strategy in the SC5314 background (Sasse and Morschhauser 2012). We confirmed by flow cytometry that no aneuploidy was introduced during the inactivation process (Figure S3A in File S2) and that no detection of *HGT16* RNA messenger was possible in the double mutant: *hgt16*Δ (Figure S3B in File S2). We then grew the SC5314 and *HGT16* mutant strains in YPD or YPM media supplemented with or without acetic acid, and measured growth rates over a 6 hr period. By qRT-PCR, we confirmed the observation made with RNA-seq, that *HGT16* expression is significantly increased by at least a fourfold factor by acetic acid when assayed in YPM media (Figure S3, A and B in File S2), but constant between YPD and YPM in the absence of acetic acid. As expected by its low level of expression in YPD and YPM alone, *HGT16* inactivation did not significantly alter growth rates between those conditions. However, the *HGT16* mutant displayed a fivefold increase in acetic acid resistance compared to the control strain in YPM complemented with acetic acid, while an increase of 33% was observed with YPD media, but this was not significant (Figure 4). These results thus confirm that *HGT16* upregulation during WOA exposure might contribute to WOA sensitivity in *C. albicans*.

## Discussion

Natural niches of *C. albicans* are complex environments where nutrient composition and availability are unpredictable and competition with other organisms for resources is strong (GI tract and skin). Mechanisms of adaptation to these conditions are critical for the yeast’s survival (Miramon and Lorenz 2017). Indeed, *C. albicans* has been described to respond to a large array of stimuli from carbon dioxide (Cottier *et al.* 2012) to substrate topography (Brand *et al.* 2008), passing by a large range of molecules like pheromones, carbon sources, or ammonium (Cottier and Muhlschlegel 2009). Most of these studies were focused on a single perturbation to the media of culture, while *C. albicans* most likely faces several changes concomitantly in its natural environment (*e.g.*, the human diet). Integration of these different and sometimes contradictory signals remains an important part of understanding the mechanisms behind the adaptability of *C. albicans* to its environment. Here, by studying the *C. albicans* response to WOAs in different media and genetic backgrounds, we aimed to identify the core components of this yeast sensitivity to WOAs. Our previous work (Cottier *et al.* 2015b) pointed out conditions where sensitivity to WOAs is enhanced without changing acid concentrations but by acting on other parameters such as carbon source (maltose instead of glucose) or the inactivation of genes (*MIG1*).

The glucose response of *C. albicans* has been described by (Rodaki *et al.* (2009); the authors performed transcriptional profiling of yeast grown in lactate compared to a range of glucose levels, from 0.01 to 1.0%, by microarray analysis (Rodaki *et al.* 2009). Similarly, we observed an increase in genes related to aerobic respiration in the absence of glucose, but from the 180 genes (expression ratio ≥ 2) identified as regulated between lactate and 1% glucose, only 20 were commonly present in our analysis. While both studies used YPD as the base medium, the strain of *C. albicans* (THE1 *vs.* SC5314), the time of incubation (30 min *vs.* 6 hr), the temperature (30° *vs.* 37°), and the alternative carbon source (lactate *vs.* maltose) were all different. Those differences could explain the lack of concordance between the two approaches. We reached a similar situation when comparing our transcriptional response of *C. albicans* to acetic and butyric acid in MRS and YPD media. Strains and conditions of culture were identical between the current study and our previous dataset (Cottier *et al.* 2015a), but we noticed only a few common responses like ATP synthesis or arginine biosynthesis, and dramatic differences regarding ribosome biogenesis. In fact, 61% of all *C. albicans* genes were significantly regulated between MRS and YPD alone, both rich media with 2% glucose, in a SC5314 strain. This high level of gene expression alteration between control samples explained the limited overlap between transcriptional profiles of *C. albicans* in response to WOAs.

Our transcriptional profile on *MIG1* inactivation presented closer similarities with previous studies. A macroarray covering 2002 out of the 6218 open reading frames from *C. albicans* was used by Murad *et al.* (2001), where they described that 0.8% of the genes (fold change ≥ 3) were regulated by Mig1p in YPD media at 30°. In our study, using an SC5314 background strain at 37°, we reported 0.74% of the total genes to be similarly regulated. Some of the principal expressed genes were identified in both studies (*HGT1*, *GCA1*, *GAL1*, *orf19.11*, *orf19.6888*, *PHR1*, and *MDH1*). We also confirmed derepression of a major gluconeogenesis enzyme, the phosphoenolpyruvate carboxykinase *PCK1*, in the *mig1*Δ strain, as previously described (Murad *et al.* 2001). In agreement with the described function of Mig1p in *S. cerevisiae* (Garcia-Salcedo *et al.* 2014), we reported that inactivation of *MIG1* led to a general derepression of genes involved in nonglucose utilization, like monosaccharide transporters (*GAL1*, *HGT1*, and *HGT16*).

From the 90 genes significantly regulated by Mig1p, we identified 36 that were also differently expressed in the presence of acetic or butyric acid in YPD media (Figure 2C). To further reduce the number of putative Mig1p targets involved in WOA sensitivity, we compared the transcriptional profiles of genes differently regulated between YPD and YPM media in the presence of acids. We noticed that in the presence of YPM, where Mig1p is inactive, the *C. albicans* SC5314 strain displayed a higher sensitivity to WOAs compared to YPD media. Similarly, an increase sensitivity to WOAs was observed between SC5314 and the *MIG1* inactivated strain in YPD media. We ended with a list of six genes (*GLG2*, *orf19.7566*, *ALD6*, *FDH1*, *HGT16*, and *orf19.94*) in which expression was negatively correlated with cell growth rate. None of these genes were part of the core stress response to WOAs that was previously identified (Cottier *et al.* 2015a), confirming the absence of a general stress response in *C. albicans*, as demonstrated by Enjalbert *et al.* (2003). Our hypothesis that inactivation of one of these genes would induce a higher resistance to WOAs was validated with *HGT16*. The mutant strain remained sensitive to WOA exposure, but showed a fivefold increase in resistance to acetic acid compared to the SC5314 wild-type strain in YPM media. The question of how to assess the remaining Mig1p targets for their role in WOA resistance persists. *Orf19.7566*, encoding another predicted membrane transporter would be a prime choice, following the results obtained with *HGT16*. We could hypothesize that these membrane transporters might open channels for the WOA molecules to be transported inside the cells, increasing their impacts on metabolic pathways.

In this study, we demonstrated an ability to reduce *C. albicans* growth rate by altering cellular compounds (*MIG1* mutant) or external cues (carbon source). We also demonstrated that the expression of certain proteins like Hgt16p is, at least partially, responsible for *C. albicans* sensitivity to WOAs. Mechanisms to target the increased expression of such genes, or alterations of carbon source in the *C. albicans* environment, might be promising methods to keep yeast growth and colonization under control.

## Supplementary Material

Supplemental material is available online at www.g3journal.org/lookup/suppl/doi:10.1534/g3.117.300238/-/DC1.
