# Supplementary material for: The Transcriptional Response of Candida albicans to Weak Organic Acids, Carbon Source, and MIG1 Inactivation Unveils a Role for HGT16 in Mediating the Fungistatic Effect of Acetic Acid
Source: G3 (Bethesda). 2017 Sep 6;7(11):3597–604. doi: 10.1534/g3.117.300238 (PMC5677169; doi:10.1534/g3.117.300238)
Supplement: Supplementary file 2 [file 3597FileS2.docx]

**The Transcriptional Response of Candida albicans to Weak Organic Acids, Carbon Source, and *MIG1* Inactivation Unveils a Role for *HGT16* in Mediating the Fungistatic Effect of Acetic Acid**

Fabien Cottier^1^, Alrina Shin Min Tan, Marina Yurieva^2^, Webber Liao, Josephine Lum, Michael Poidinger, Francesca Zolezzi^3^, Norman Pavelka^1^

Singapore Immunology Network (SIgN), Agency for Science, Technology and Research (A*STAR), Singapore 138648, Singapore

^1^Corresponding authors: Singapore Immunology Network (SIgN), Agency for Science, Technology and Research (A*STAR), 8A Biomedical Grove, Immunos #04, Singapore 138648, Singapore. E-mail: [cottier.fabien@gmail.com](mailto:cottier.fabien@gmail.com); [norman_pavelka@immunol.a-star.edu.sg](mailto:norman_pavelka@immunol.a-star.edu.sg)

^2^Present address: The Jackson Laboratory for Genomic Medicine, Farmington, CT 06032.

^3^Present address: GALDERMA R&D, 06902 Sophia Antipolis Cedex, France.

**Additional Materials and Methods**

**Construction of *C. albicans* mutant strains**

*HGT16* alleles inactivation was performed using *SAT1*-flipping strategy in a similar manner as previously published ([Cottier et al. 2015b](#_ENREF_2)). A plasmid containing the *SAT1* flipper cassette was framed with upstream and downstream sequence of the *HGT16* gene of *C. albicans*. Flanking sequences of the *HGT16* gene (NCBI Reference Sequence: XP_712348.2) were first amplified from SC5314 genomic DNA by polymerase chain reaction (PCR) with primers (HGT16upF-KpnI, HGT16upR-ApaI, HGT16doF-SacII and HGT16doR-SacI) as listed in Table S6. Proper gene inactivation was confirmed by quantitative RT-PCR (Fig. 7).

## Ploidy analysis by flow cytometry

Relative DNA content of *HGT16* mutants were assessed using a similar protocol as previously published ([Cottier et al. 2015b](#_ENREF_2)).

**Quantitative real-time PCR analysis**:

RNA extraction for validation of transcript expression was performed according to a previously published protocol ([Cottier et al. 2015a](#_ENREF_1)) using primers presented in Table S6.


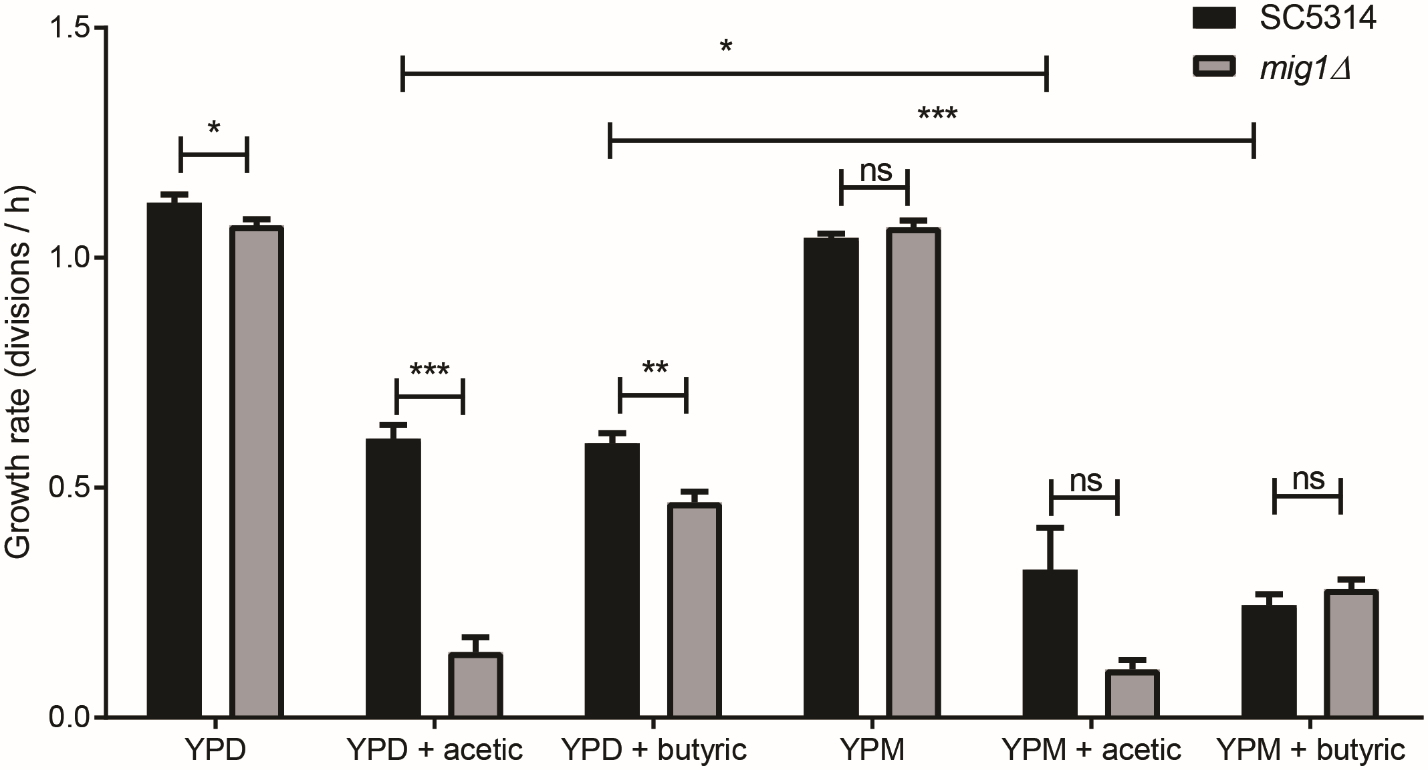


**Figure S1.** Quantitative growth assays of control (SC5314) and *mig1*Δ strain performed in YPD or YPM supplemented with acetic or butyric acid, at their respective IC_50_ value and adjusted to pH 5.5. Incubation were performed at 37 °C, and growth rate were calculated by monitoring O.D.600nm over a period of 6 hours. Data is represented as mean and SEM of 4 biological replicates. *** p < 0.001, ** p < 0.01; * p < 0.05.


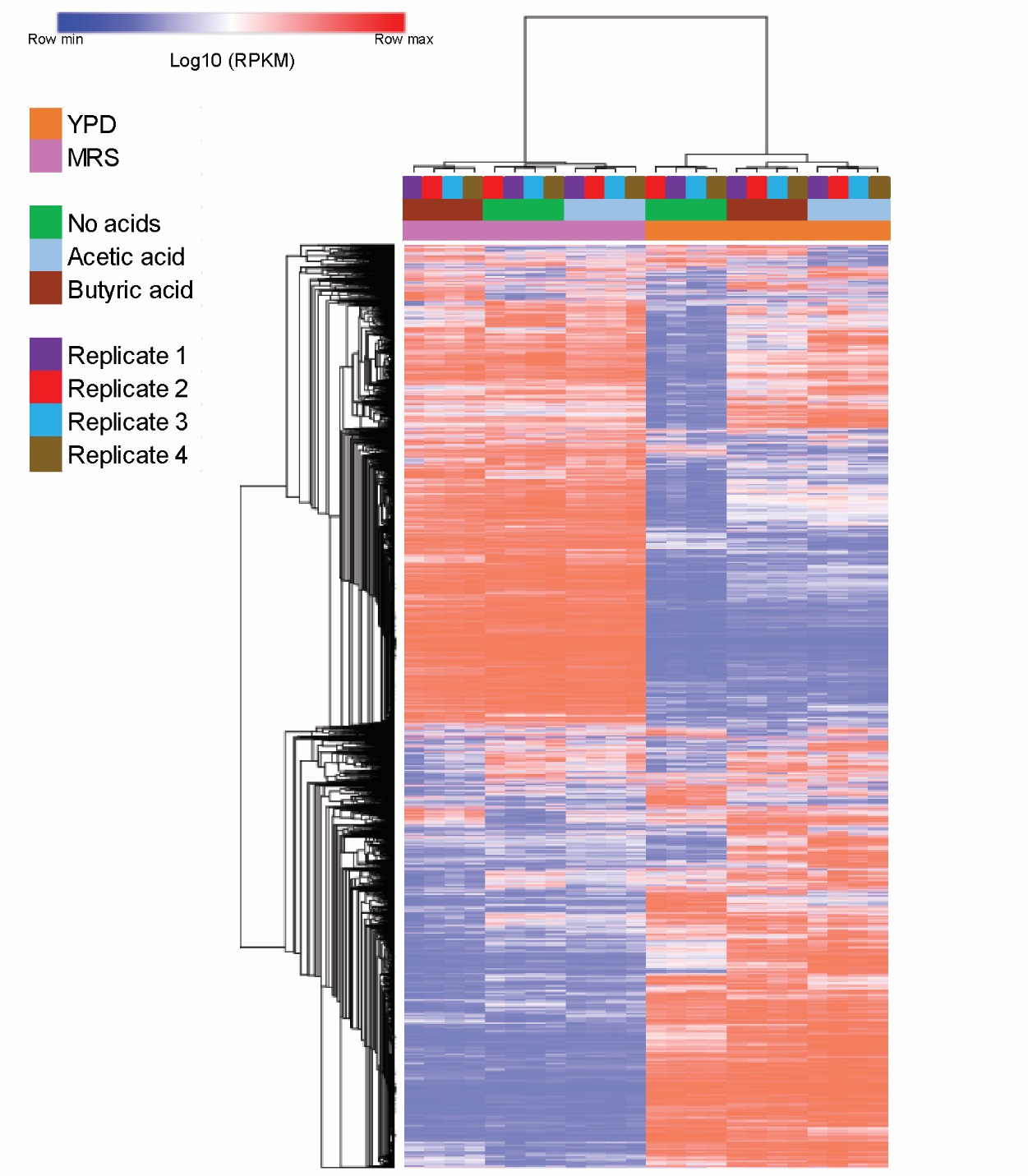


## Figure S2. Global transcriptional response of *C. albicans* to MRS and YPD media supplemented with acetic and butyric acid. The figure displays a two-way hierarchical clustering of 6,218 transcripts (rows) and 24 samples (columns). Log10-transformed RPKM expression values were converted to z-scores, with red indicating expression levels above and blue symbolizing expression levels below the mean expression level of each gene across the samples. Distance metric: 1 − Pearson correlation.

**
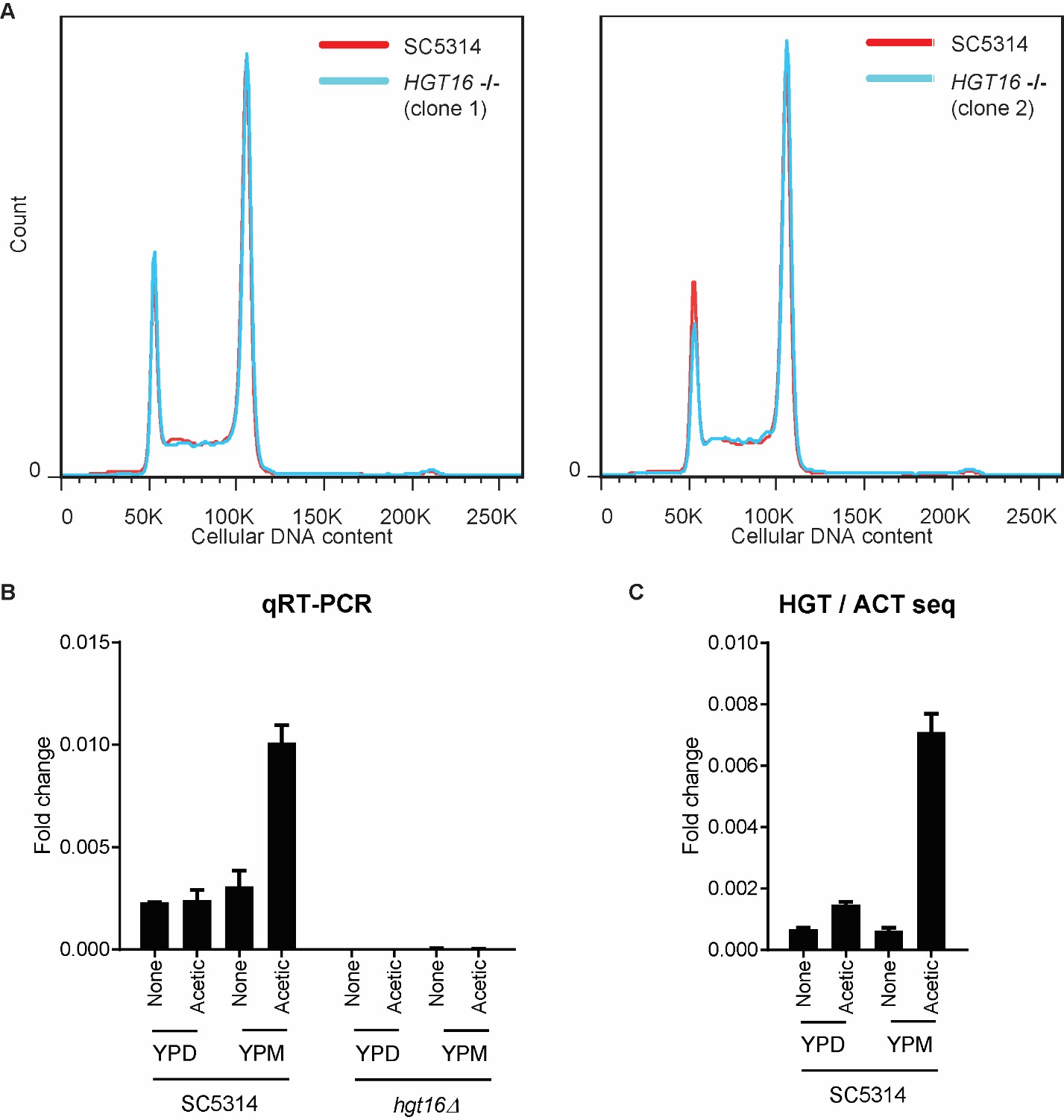
**

*

*

*

**Figure S3.** (**A**) Absence of aneuploidy in *hgt16Δ* strain. Analysis of cellular DNA content by flow cytometry from WT and two independent *hgt16Δ* mutant strains show no visible differences. (**B**) Fold change of *HGT16* over *ACT1* messengers obtained by qRT-PCR in indicated media for SC5314 and *hgt16Δ* strains. As expected, no *HGT16* mRNA was detected in any condition for *HGT16* mutant. (**C**) Ratio of RPKM value of *HGT16* over *ACT1* obtained by RNA-sequencing in indicated media for SC5314. * p < 0.05

**Table S1. Open-reading frames and non-coding RNAs significantly regulated in response to carbon source.** Genes differentially expressed in response to maltose over glucose in SC5314 and *mig1Δ* strain. These lists were used to draw the diagram in Figure 2A. Transcript identifiers refer to the *C. albicans* Assembly 21 genome annotation.

| **Condition** | **Genes significantly regulated in the same direction at all time-points** |
| --- | --- |
| Up-regulated only in SC5314 | *orf19.6117, orf19.6898.1, orf19.5095, orf19.1339, orf19.3940.1, orf19.4953, orf19.3757, orf19.2297, orf19.5713, orf19.5114, orf19.5267, orf19.3782.2, orf19.7210, orf19.3105, orf19.673, orf19.6492, orf19.347, orf19.3373, orf19.7527, orf19.6531, orf19.2284, orf19.2881, orf19.3198, orf19.1562, orf19.3543, orf19.4304, orf19.4122, orf19.697, orf19.255, orf19.6307, orf19.2747, orf19.5893, snR8b, orf19.3679, orf19.5843, orf19.1974, orf19.6645, orf19.6165, orf19.669, orf19.338, orf19.6791, orf19.868, orf19.7214, orf19.2769, orf19.7316, orf19.1978, orf19.4885, orf19.1266, orf19.7379, orf19.2693, orf19.4432, orf19.6077, orf19.6550, orf19.5622, orf19.4416, orf19.1183, orf19.4174, orf19.5294, orf19.6073, CaalfMr16, orf19.3004, orf19.6881, orf19.4151, orf19.3742, orf19.6887, orf19.3097, orf19.3131, orf19.873.1, orf19.6366, orf19.2067, orf19.1107, orf19.3223.1, orf19.1799, orf19.4816, orf19.3611, orf19.1891, orf19.7319, orf19.6658, orf19.846, orf19.802, orf19.2050, orf19.2655, orf19.4199, orf19.5280, orf19.4602, orf19.4883, orf19.5102, orf19.6794, orf19.5730, orf19.3544, orf19.5813, orf19.1069, orf19.6877, orf19.4608, orf19.703, orf19.5204, orf19.6644, orf19.7114, orf19.6478, orf19.7551, orf19.1075.1, orf19.4543, orf19.5064.1, orf19.6709, orf19.2204, orf19.1153, orf19.4370, orf19.6592, orf19.5806, orf19.7242, orf19.1800, orf19.2841, orf19.2838, orf19.2812, orf19.1862, orf19.899, orf19.2219, orf19.6706, orf19.2529.1, orf19.6724, orf19.4941, orf19.4980, orf19.7281, orf19.1584, orf19.1863, orf19.4979, orf19.1719, orf19.6061, orf19.2809, orf19.5597.1, orf19.7666, orf19.4759, CaalfMr17, orf19.2077, orf19.3133, orf19.415, orf19.3139, orf19.2414, orf19.733, orf19.2282, orf19.575, orf19.1375, orf19.6196, orf19.2076, orf19.4132, orf19.7472, orf19.1395, orf19.913.2, orf19.6947, orf19.287, orf19.4490.2, orf19.7227, orf19.6720, orf19.5079.1, orf19.7225, orf19.4679, orf19.1764, orf19.6132, orf19.5553, orf19.4229, orf19.7568, orf19.1872, orf19.2707.1, orf19.286, orf19.6387, orf19.4495, orf19.1367.1, orf19.4090, orf19.842, orf19.6474, orf19.1156, orf19.3527, orf19.5961, orf19.1861, orf19.5124, orf19.6578, orf19.5077, orf19.227, orf19.4795, orf19.4317, orf19.2023, orf19.4946, orf19.853, orf19.5759, orf19.5205, orf19.4901, orf19.3579, orf19.4751.1, orf19.1785, orf19.6245, orf19.5282, orf19.750, orf19.2726, orf19.4826, orf19.918, orf19.3863, orf19.7509.1, orf19.7590, orf19.2079, orf19.86, orf19.7311, orf19.2306, orf19.5547, orf19.2613, orf19.2108, orf19.100, orf19.542, orf19.3351, orf19.1656, orf19.2972, orf19.4940, orf19.2066.1, orf19.6385, orf19.1471, orf19.6308, orf19.2082, orf19.5295, orf19.6449, orf19.3827, orf19.6637, orf19.5960, orf19.3160, orf19.1193, orf19.2439.1, orf19.2896, orf19.5958, orf19.4607, orf19.7437, orf19.2030, orf19.3434, orf19.35, orf19.1873, orf19.2954, orf19.3674, orf19.1523, orf19.4856, orf19.1826, orf19.446.2, orf19.2458, orf19.3661, orf19.4012, orf19.925, orf19.5159, orf19.4792, orf19.1082.1, orf19.10, orf19.5791, orf19.2839, orf19.332.1, orf19.6586, orf19.2242, orf19.5720, orf19.1473, orf19.2395, orf19.3310, orf19.5653, orf19.7209, orf19.1150.1, orf19.5137, orf19.5158, orf19.6659, orf19.4791, orf19.97, orf19.2608, orf19.2770, orf19.6660, orf19.4821, orf19.3675, orf19.4947, orf19.882, orf19.4743, orf19.5288, orf19.3311, orf19.6229, orf19.4970, orf19.5079, orf19.4121, orf19.6000, orf19.7490, orf19.4898, orf19.4894, orf19.111, orf19.6793, orf19.6888, orf19.2959.1, orf19.5125, orf19.2748, orf19.5076.1, orf19.2737, orf19.1152, orf19.2168, orf19.2531, orf19.2107.1, orf19.2020, orf19.4476, orf19.873, orf19.1655.3, orf19.3612, orf19.6941, orf19.4211, orf19.1586, orf19.1321, orf19.2048, orf19.3707, orf19.4017, orf19.2021, orf19.5805, orf19.5626, orf19.3439, orf19.265, orf19.7093, orf19.3684, orf19.5611, orf19.5114.1, orf19.3932.1, orf19.2376, orf19.6311, orf19.7029, orf19.2515, orf19.7514, orf19.3369, orf19.4384, orf19.1354, orf19.1353, orf19.94, orf19.638, orf19.3670, orf19.4384.1, orf19.6391, orf19.3499, orf19.2620, orf19.2701, orf19.11, orf19.823, orf19.4445, orf19.5447, orf19.6084, orf19.3672, orf19.6688, orf19.4773, orf19.7434, orf19.3982, orf19.732, orf19.2870, orf19.4936.1, orf19.4450.1, orf19.5975, orf19.2897, orf19.4682, orf19.4899, orf19.4526* |
| Down-regulated only in SC5314 | *tY(GUA)3, orf19.2652, orf19.6998, orf19.4169, orf19.4853, orf19.95, orf19.2451, orf19.3761, orf19.2657, orf19.7634, orf19.658, orf19.2885, orf19.5811, orf19.5487, orf19.1168, orf19.687, orf19.4354, orf19.262, orf19.5025, orf19.403, orf19.4069, orf19.1720, orf19.7190, orf19.1606, orf19.4690, orf19.6873.1, orf19.7564, orf19.2611, orf19.4367, orf19.2452, orf19.2796, orf19.4136, orf19.1445, orf19.5779, orf19.4669, orf19.1901, orf19.4583, orf19.1828, orf19.2649, orf19.2417, orf19.4255, orf19.6784, orf19.6398, orf19.4945, orf19.5012, orf19.6277, orf19.4161, orf19.5126, orf19.1631, orf19.202, orf19.1782.1, orf19.5873, orf19.1825, orf19.938, orf19.5166, orf19.1673, orf19.2478, orf19.6026, orf19.2739, orf19.4447, orf19.6568, orf19.6022, orf19.926, orf19.1510, orf19.5934, snR39b, orf19.4301, orf19.1442, orf19.4616, orf19.3378, orf19.768, orf19.4080, orf19.4765, orf19.6896, orf19.3466, orf19.6247, orf19.3207, orf19.3780, orf19.234, orf19.2738, orf19.120, orf19.4215, orf19.7494, orf19.5839, orf19.3538, orf19.4055, orf19.3740, orf19.1836, orf19.4076, orf19.7675, orf19.2216, orf19.5169, orf19.3322, orf19.663, orf19.7017, orf19.2673, orf19.5191, orf19.547, orf19.5043, orf19.5674, orf19.3282, orf19.3549, orf19.6086, orf19.2650.1, orf19.6662, orf19.5645, orf19.7658, orf19.5861.1, orf19.6972, orf19.3721, orf19.7394, orf19.4162, orf19.568, orf19.3751, orf19.946, orf19.7065, orf19.4030, orf19.3100, orf19.4890, orf19.5242, orf19.789.1, orf19.6907, orf19.4664, orf19.1115, orf19.4142, orf19.6186, orf19.4809, orf19.4261, orf19.7517, orf19.3423, orf19.6562, orf19.5564, orf19.3140, orf19.1835, orf19.5762, orf19.6569, orf19.6864, orf19.4712, orf19.1626, orf19.7202, orf19.1351, orf19.7232, orf19.7456, orf19.1605, orf19.2311, orf19.7083, orf19.3380, orf19.5244, orf19.3468, orf19.5180, orf19.6345, orf19.5694, orf19.3501, orf19.244, orf19.7293, orf19.1697, orf19.3406, orf19.1598, orf19.1834, orf19.6286.2, orf19.6105, orf19.4355, orf19.85, orf19.203, orf19.506, orf19.4563, orf19.4099, orf19.1447, orf19.7263, orf19.3255, orf19.2544, orf19.2875, orf19.2365, orf19.727, orf19.5792, orf19.5693, orf19.3536, orf19.5992, orf19.5919, orf19.1700, orf19.676, orf19.1746, orf19.3920, orf19.1757, orf19.3916, orf19.6829, orf19.1964, orf19.6220.4, orf19.7354, orf19.4725, orf19.3626, orf19.6402, orf19.993, orf19.3715, orf19.2465, orf19.5775, orf19.6610, orf19.7298, orf19.5641, orf19.5921, orf19.7621, orf19.3394, orf19.2382, orf19.6328, orf19.3205, orf19.6100, orf19.3265, orf19.3481, orf19.7082, orf19.4223, orf19.6343, orf19.6565, orf19.4506, orf19.1823, orf19.4647, orf19.6785, orf19.7127.1, orf19.4308, orf19.1409.1, orf19.3490, orf19.4962, orf19.1691, orf19.3581, orf19.5488, orf19.1391, orf19.7452, orf19.2930, orf19.6526, orf19.837, orf19.5009, orf19.2310, orf19.3124, orf19.657, orf19.4279, orf19.4517, orf19.7301, orf19.7312, orf19.1618, orf19.6584, orf19.7521, orf19.6975, orf19.4336, orf19.6294, orf19.7327, orf19.6786, orf19.5466, orf19.4051, orf19.4805, orf19.6579, orf19.6851, orf19.5020, orf19.2775, orf19.2639, orf19.3788.1, orf19.660, orf19.668, orf19.1265, orf19.828, orf19.4831, orf19.6227, orf19.4635, orf19.6291, orf19.5949, orf19.4283, orf19.4349, orf19.5801, orf19.1146, orf19.3214, orf19.4459, orf19.236, orf19.7360, orf19.5928, orf19.3061.1, orf19.1167, orf19.4880, orf19.5341, orf19.4931, orf19.3211, orf19.7328, orf19.7425, orf19.3206, orf19.1234, orf19.2919, orf19.3334, orf19.5648, orf19.4688, orf19.6558, orf19.3048, orf19.7121, orf19.1361, orf19.6219, orf19.4963, orf19.3138, orf19.6477, orf19.2798, orf19.3219, orf19.1716, orf19.5510, orf19.4125, orf19.4376, orf19.406, orf19.5368, orf19.3266, orf19.6027, orf19.7401, orf19.5802, orf19.3503, orf19.688, orf19.2259, orf19.5056, orf19.2873, orf19.708, orf19.5433, orf19.2065, orf19.4257, orf19.7610, orf19.3960, orf19.3099, orf19.3370, orf19.1441, orf19.7588, orf19.7236, orf19.7548, orf19.6541, orf19.2994, orf19.5698, orf19.5271, orf19.4662, orf19.1948, orf19.3797, orf19.3680, orf19.3603, orf19.3202, orf19.1755, orf19.7260, orf19.4435, orf19.3216, orf19.3561, orf19.923, orf19.5335, orf19.7358, orf19.4190, orf19.4660, orf19.2837, orf19.5809, orf19.1208, orf19.2641, orf19.1326, orf19.3001, orf19.5399, orf19.6694, orf19.5071, orf19.4002, orf19.2212, orf19.7538, orf19.6129, orf19.2098, orf19.7217, orf19.1969, orf19.4167, orf19.854, orf19.5015, orf19.4966, orf19.1600, orf19.1295, orf19.3753, orf19.2992, orf19.7409.1, orf19.2087, orf19.2952, orf19.3865, orf19.3928, orf19.6417, orf19.2350, orf19.2475, orf19.6265.1, orf19.7019, orf19.680, orf19.1533, orf19.3526, orf19.3438, orf19.1192, orf19.2496, orf19.6282, orf19.1613, orf19.3396, orf19.5013, orf19.5051, orf19.6025, orf19.4931.1, orf19.5541, orf19.7009, orf19.427, orf19.6124, orf19.5996.1, orf19.2213, orf19.5518, orf19.7377, orf19.6265, orf19.1967, orf19.5495, orf19.7012, orf19.1359, orf19.6813, orf19.2601, orf19.3798, orf19.4312, orf19.5141, orf19.3087, orf19.5650, orf19.1662, orf19.5081, orf19.2560, orf19.5143, orf19.4889, orf19.7157, orf19.4022, orf19.5929, orf19.1144, orf19.1923, orf19.1137, orf19.7265, orf19.4021, orf19.6002, orf19.1747, orf19.5050, orf19.863, orf19.7221, orf19.7079, orf19.6156, orf19.4208, orf19.3051, orf19.4377, orf19.1414, orf19.2179.2, orf19.7353, orf19.2830, orf19.943, orf19.2238, orf19.2533.1, orf19.198, orf19.431, orf19.3240, orf19.3942, orf19.3470, orf19.5035, orf19.3803, orf19.5595, orf19.1064, orf19.3648, orf19.1782, orf19.1559, orf19.5576, orf19.5927, orf19.1589.1, orf19.2237.1, orf19.96, orf19.1832, orf19.4064, orf19.7357, orf19.1966, orf19.7362, orf19.6783, orf19.6375, orf19.2093, orf19.24, orf19.1116, orf19.5578, orf19.3558, orf19.5704, orf19.4149, orf19.3676, orf19.6748, orf19.585, orf19.5316, orf19.7337, orf19.2287, orf19.755, orf19.6705, orf19.1426, orf19.5964.2, orf19.6792, orf19.3034, orf19.4176, orf19.3003.1, orf19.3487, orf19.2937, orf19.7050, orf19.1988, orf19.5474, orf19.1738.1, orf19.979, orf19.5692, orf19.6456, orf19.4490, orf19.88, orf19.2859, orf19.4659, orf19.2389, orf19.1630, orf19.6205, orf19.1667.1, orf19.557, orf19.6312, orf19.3736, orf19.6679, orf19.2111.2, orf19.7161, orf19.3934, orf19.6008, orf19.1169, orf19.5483, orf19.2721, orf19.646, orf19.5465, orf19.3965, orf19.3305, orf19.564, orf19.5517, orf19.1614, orf19.7413, orf19.4747, orf19.2994.1, orf19.5182, orf19.2512, orf19.6293, orf19.6109, orf19.7313, orf19.231, orf19.6085, orf19.3693, orf19.5216, orf19.7102, orf19.150, orf19.3831, orf19.805, orf19.4212, orf19.3534, orf19.2606, orf19.3504, orf19.667.1, orf19.7577, orf19.1170, orf19.6010, orf19.1390, orf19.4666, orf19.2167, orf19.4787, orf19.5243, orf19.7194, orf19.641, orf19.2305, orf19.2128, orf19.7561, orf19.5083, orf19.2479, orf19.4152, orf19.2927, orf19.1635, orf19.5225.2, orf19.2119, orf19.6749, orf19.5147, orf19.7047, orf19.2960, orf19.3461, orf19.7203, orf19.7049, orf19.1334, orf19.6921, orf19.2546, orf19.3083, orf19.6670, orf19.102, orf19.5834, orf19.1232, orf19.6177, orf19.2000, orf19.2651, orf19.4149.1, orf19.5685, orf19.6587, orf19.665, orf19.4670, orf19.4733, orf19.3541, orf19.5841, orf19.7266, orf19.6041, orf19.1569, orf19.7018, orf19.2309.2, orf19.7291, orf19.840, orf19.5469, orf19.1522, orf19.7035, orf19.7613, orf19.7645, orf19.6906, orf19.57, orf19.4060, orf19.7477, orf19.1917, orf19.2746, orf19.3690.2, orf19.7153, orf19.397, orf19.3477, orf19.2322.3, orf19.7219, orf19.498, orf19.22, orf19.2415, orf19.5103, orf19.2422, orf19.7614, orf19.6696, orf19.4109, orf19.6403.1, orf19.712, orf19.6420, orf19.3080, orf19.484, orf19.7665, orf19.2060, orf19.3449, orf19.3599, orf19.3350, orf19.2303, orf19.7402, orf19.3465, orf19.6882.1, orf19.5982, orf19.4665, orf19.4701, orf19.1668, orf19.6191, orf19.18, orf19.3367, orf19.1143, orf19.2478.1, orf19.3559, orf19.2740, orf19.210, orf19.163, orf19.1934, orf19.3942.1, orf19.4341, orf19.5211, orf19.6213, orf19.6326, orf19.3126, orf19.1973, orf19.7537, orf19.7223, orf19.6346, orf19.2720, orf19.4004, orf19.6013, orf19.5691, orf19.5918, orf19.2864.1, orf19.5376, orf19.2183, orf19.4265, orf19.6202, orf19.687.1, orf19.3002, orf19.2399, orf19.4580, orf19.3551, orf19.7615, orf19.5041, orf19.6392, orf19.6873, orf19.2309, orf19.6133, orf19.3471, orf19.2029, orf19.401, orf19.3318, orf19.5502, orf19.4675, orf19.4718, orf19.4079, orf19.1417, orf19.7048.1, orf19.4061, orf19.7604, orf19.5917.3, orf19.832, orf19.2275, orf19.6804, orf19.6099, orf19.7650, orf19.1039, orf19.122, orf19.6885, orf19.5085, orf19.5875, orf19.532, orf19.2360, orf19.493, orf19.1536, orf19.714, orf19.4193.1, orf19.407, orf19.4068, orf19.6264.4, orf19.1494, orf19.5747, orf19.6295, orf19.4628, orf19.2246, orf19.4796, orf19.2597, orf19.5369, orf19.4600.1, orf19.6438, orf19.3057, orf19.1791, orf19.5444, orf19.7270, orf19.2088, orf19.2767, orf19.4324, orf19.1031, orf19.4632, orf19.6184, orf19.4751, orf19.6855, orf19.2760, orf19.5420, orf19.2709, orf19.587, orf19.5838, orf19.269, orf19.6136, orf19.1609, orf19.7466, orf19.3572.3, orf19.3795, orf19.2991, orf19.6057, orf19.6419.1, orf19.6217, orf19.7175, orf19.7648, orf19.3474, orf19.3415.1, orf19.7448, orf19.7609, orf19.5066, orf19.2320, orf19.4036, orf19.1666, orf19.5287, orf19.5377, orf19.6663, orf19.7057, orf19.6858, orf19.2715, orf19.7516, orf19.3239, orf19.7375, orf19.2329.1, orf19.4937, orf19.2286, orf19.827.1, orf19.5230, orf19.7368, orf19.4457* |
| Up-regulated in SC5314 and *mig1Δ* | *orf19.5785, orf19.2270, orf19.7231, orf19.6139, orf19.238, orf19.1149, orf19.3364, orf19.4774, orf19.4943, orf19.4477, orf19.6951, orf19.7586, orf19.2968, orf19.2091, orf19.3104, orf19.7306, orf19.5629, orf19.4396, orf19.6062.3, orf19.4216, orf19.1682, orf19.7085, orf19.847, orf19.4016, orf19.5419, orf19.5686, orf19.2157, orf19.1331, orf19.2821, orf19.6322, orf19.2241, orf19.5660.1, orf19.7111.1, orf19.5683, orf19.251, orf19.1830, orf19.3223, orf19.5213.2, orf19.6854, orf19.1890, orf19.2768, orf19.5213.1, orf19.419, orf19.3340, orf19.2785, orf19.5491.1, orf19.3932, orf19.3507, orf19.5070, orf19.7284, orf19.2296, orf19.5201.1, orf19.5610, orf19.449, orf19.1467, orf19.804.1, orf19.2644, orf19.3770, orf19.4527, orf19.3335, orf19.2724, orf19.7310, orf19.2849, orf19.7469, orf19.2344, orf19.3548.1, orf19.6489, orf19.5005, orf19.6514, orf19.5000, orf19.822, orf19.1048, orf19.5565, orf19.7283, orf19.1449, orf19.4914.1, orf19.1363, orf19.3664, orf19.670.2, orf19.999, orf19.3981, orf19.7668, orf19.4886* |
| Down-regulated in SC5314 and *mig1Δ* | *tG(GCC)6, orf19.689, orf19.6570, orf19.5673, orf19.5842, orf19.5870, orf19.1264, orf19.2602, orf19.1113, orf19.6222.1, orf19.4105, orf19.4170, orf19.5784, orf19.1702, orf19.4802, orf19.2584, orf19.496, orf19.576, orf19.386, orf19.5226, orf19.4560, orf19.7188, orf19.1065, orf19.6936, orf19.7631, orf19.385, orf19.2232, orf19.6632, orf19.3106, orf19.1601, orf19.4545, orf19.2179, orf19.417, orf19.1591, orf19.245, orf19.5905, orf19.84, orf19.1986, orf19.1405, orf19.7015, orf19.6072, orf19.5367, orf19.4716, orf19.2525* |
| Up-regulated only in *mig1Δ* | *orf19.6948, orf19.3107, snR189c* |
| Down-regulated only in *mig1Δ* | *snR78, tE(UUC)3, orf19.6110, orf19.2583.2, orf19.7002, orf19.5831, orf19.4933, orf19.5755* |

**Table S2. Open-reading frames and non-coding RNAs significantly regulated in response to WOAs.** Genes differentially expressed in response to acetic or butyric acid exposure in YPD media for SC5314 strain. These lists were used to draw the diagram in Figure 2B. Transcript identifiers refer to the *C. albicans* Assembly 21 genome annotation.

| **Condition** | **Genes significantly regulated in the same direction at all time-points** |
| --- | --- |
| Up-regulated in acetic acid only | *orf19.1717, orf19.6610, orf19.1584, orf19.2444, orf19.4398, orf19.1967, orf19.4250, orf19.6202, orf19.5845, orf19.2236, orf19.4432, orf19.5068, orf19.5589, orf19.7527, orf19.6366, orf19.961.2, orf19.5168, orf19.2921, orf19.6779, orf19.6008.4, orf19.2272, orf19.7457, orf19.4362, orf19.115, orf19.2953, orf19.2178, orf19.7068, orf19.1382, orf19.3938, orf19.2876, orf19.674, orf19.4905, orf19.300, orf19.5930, orf19.7645, orf19.3362, orf19.7125, orf19.1064, orf19.5534, orf19.5052, orf19.6727, orf19.4892, orf19.1179, orf19.6676, orf19.4051, orf19.5360, orf19.1164, orf19.875, orf19.6900, orf19.3912, orf19.2198, orf19.4898, orf19.916, orf19.6052, orf19.4643, orf19.4306, orf19.3693, orf19.3012, orf19.967, orf19.1800, orf19.1195, orf19.2043, orf19.2809, orf19.3705, orf19.3704.1, orf19.7053, orf19.4807, orf19.150, orf19.4963, orf19.5557, orf19.7489.3, orf19.4127, orf19.7488, orf19.5251, orf19.4967, orf19.2455, orf19.6583, orf19.3556, orf19.679, orf19.2770.1, orf19.6477, orf19.1669, orf19.4924, orf19.6369, orf19.6600, orf19.7657, orf19.5483, orf19.6980, orf19.3963, orf19.498, orf19.2363, orf19.549, orf19.585, orf19.4496, orf19.6979, orf19.3132, orf19.5832, orf19.4263, orf19.2124, orf19.25, orf19.240, orf19.7029, orf19.4929, orf19.5967, orf19.458, orf19.7409.1, orf19.1662, orf19.151, orf19.7229, orf19.425, orf19.5230, orf19.2042, orf19.5517, orf19.2330, orf19.2830, orf19.7063, orf19.7316, orf19.1191, orf19.184, orf19.2819, orf19.4869, orf19.6524, orf19.52, orf19.5663, orf19.3887, orf19.5430, orf19.4232, orf19.3367, orf19.3034, orf19.6463, orf19.1114, orf19.3415, orf19.6803, orf19.3498, orf19.4515, orf19.755, orf19.3950, orf19.17, orf19.4716, orf19.783, orf19.3691, orf19.879, orf19.3543, orf19.1767, orf19.2639, orf19.6533, orf19.3308, orf19.4467, orf19.3022, orf19.1305, orf19.1545, orf19.2459, orf19.484, orf19.4059, orf19.3859, orf19.804, orf19.5869, orf19.1927, orf19.2382, orf19.3846, orf19.3153, orf19.5215, orf19.5387, orf19.1623, orf19.439, orf19.3040, orf19.639.1, orf19.5746, orf19.6678, orf19.4001, orf19.6392, orf19.934, orf19.2623, orf19.4409, orf19.2851, orf19.947, orf19.251, orf19.2438, orf19.5867, orf19.91, orf19.5425, orf19.5877, orf19.5777, orf19.2384, orf19.3348, orf19.5381, orf19.522, orf19.7184, orf19.5960, orf19.3535, orf19.7111.1, orf19.3202, orf19.6723, orf19.2201, orf19.4702, orf19.2498, orf19.4018, orf19.5926, orf19.4271, orf19.2284, orf19.1794, orf19.2520, orf19.1776, orf19.7020, orf19.2057, orf19.6499, orf19.6596, orf19.7678, orf19.3114, orf19.6853, orf19.7130, orf19.477, orf19.2066, orf19.5610, orf19.1949, orf19.2631, orf19.4676, orf19.1797, orf19.2342, orf19.7478, orf19.4299, orf19.1981, orf19.2137, orf19.7010, orf19.1042, orf19.1546, orf19.5879, orf19.2784, orf19.6156, orf19.1396, orf19.3102, orf19.4197, orf19.4937, orf19.5677, orf19.336, orf19.1291, orf19.2183, orf19.6503, orf19.3851, orf19.675, orf19.5388, orf19.5241, orf19.4577.3, orf19.2237.1, orf19.4751, orf19.6295, orf19.7387, orf19.3205, orf19.2956, orf19.2331, orf19.4906, orf19.2721, orf19.323, orf19.1133, orf19.216.1, orf19.863, orf19.3127, orf19.5420, orf19.1415, orf19.3357, orf19.3649, orf19.3777, orf19.297, orf19.2532, orf19.3265, orf19.5639, orf19.2825, orf19.5161, orf19.5808, orf19.4464, orf19.747, orf19.4522, orf19.4527, orf19.2650.1, orf19.3501, orf19.828, orf19.7654, orf19.2275, orf19.460, orf19.6263, orf19.5751, orf19.2028, orf19.3227, orf19.4506, orf19.4630, orf19.954, orf19.4182, orf19.5720, orf19.6041, orf19.5515, orf19.6577, orf19.6431, orf19.3480, orf19.2115, orf19.4705, orf19.1528, orf19.5012, orf19.5698, orf19.4204, orf19.7370, orf19.805, orf19.4358, orf19.1624.1, orf19.4465, orf19.6113, orf19.4792, orf19.5037, orf19.7012, orf19.2984, orf19.3275, orf19.3592, orf19.3559, orf19.4028, orf19.5258, orf19.871, orf19.5541, orf19.6696, orf19.4377, orf19.3181.1, orf19.2008, orf19.1405, orf19.6829, orf19.2547, orf19.1070, orf19.5194.1, orf19.3631, orf19.2501, orf19.5599, orf19.192, orf19.4023, orf19.7019, orf19.6062, orf19.6595, orf19.4721, orf19.5167, orf19.3111, orf19.1350, orf19.5747, orf19.7603, orf19.1772, orf19.2415, orf19.7245, orf19.6632, orf19.7443, orf19.4727, orf19.7307, orf19.6972, orf19.2104, orf19.1966, orf19.6736, orf19.6136, orf19.3448, orf19.310, orf19.5255, orf19.515, orf19.3188, orf19.1727, orf19.6698, orf19.2752, orf19.4357, orf19.5235, orf19.264, orf19.2927, orf19.6192, orf19.2778, orf19.4418, orf19.3432.1, orf19.3797, orf19.445, orf19.5064, orf19.51, orf19.677, orf19.4788, orf19.2003, orf19.5814.1, orf19.3297, orf19.263.1, orf19.432, orf19.7436, orf19.4583, orf19.3782, orf19.7371, orf19.417, orf19.4097, orf19.7187, orf19.3221, orf19.2832, orf19.2039, orf19.7084, orf19.7601, orf19.6565, orf19.5705, orf19.5020, orf19.3301, orf19.1485, orf19.5716, orf19.246, orf19.3267, orf19.215, orf19.5748, orf19.1765, orf19.2387, orf19.2977, orf19.5636, orf19.2638, orf19.6653, orf19.3736, orf19.2006.1, orf19.1795, orf19.185, orf19.6482, orf19.4601, orf19.2457, orf19.6515, orf19.4574, orf19.1300, orf19.3861, orf19.2214, orf19.5966, orf19.506, orf19.6950, orf19.2511.1, orf19.69.2, orf19.6805, orf19.4980, orf19.6420, orf19.3396, orf19.3352, orf19.2754, orf19.3089, orf19.4884, orf19.2013, orf19.5611, orf19.7602, orf19.7091, orf19.1673, orf19.5722, orf19.4324, orf19.1708, orf19.6917, orf19.6532, orf19.7504, orf19.5797, orf19.326, orf19.4952, orf19.6840, orf19.1618.1, orf19.3306, orf19.4056, orf19.3538, orf19.7386, orf19.1643, orf19.6208, orf19.999, orf19.2344, orf19.6916, orf19.4190, orf19.5449, orf19.7222, orf19.1481, orf19.3526, orf19.685, orf19.23, orf19.24, orf19.909, orf19.3732, orf19.7296, orf19.7194, orf19.3644, orf19.5097, orf19.5749, orf19.6413, orf19.1728, orf19.3192, orf19.5902, orf19.345, orf19.6139, orf19.3986, orf19.717, orf19.1069, orf19.5050, orf19.6140, orf19.2897, orf19.2445, orf19.6387, orf19.5915, orf19.3406, orf19.4463, orf19.802, orf19.2509.1, orf19.1321, orf19.7585, orf19.5713, orf19.6888, orf19.7469, orf19.4872, orf19.4310, orf19.2787, orf19.344, orf19.4899, orf19.6715, orf19.22.1, orf19.3337, orf19.5606, orf19.3924, orf19.6391, orf19.6274, orf19.4921.1, orf19.6547, orf19.5741, orf19.1774, orf19.6487* |
| Down-regulated in acetic acid only | *tR(UCU)4, orf19.6350, orf19.1782.1, orf19.4914.1, orf19.6993, orf19.5141, orf19.7502, orf19.1167, orf19.4366, orf19.2762, orf19.7330, orf19.4287, orf19.5806, orf19.792, orf19.3310, orf19.2608, orf19.1121, orf19.5499, orf19.164, orf19.915, orf19.4539, orf19.5525, orf19.1048, orf19.1354, orf19.411, orf19.750, orf19.3419, orf19.3997, orf19.5113, orf19.7218, orf19.6745, orf19.395, orf19.5136, orf19.4279, orf19.1067, orf19.6282, orf19.2107.1, orf19.768, orf19.113, orf19.1353, orf19.111, orf19.787.1, orf19.5348, orf19.550, orf19.2681, orf19.6882, orf19.7027, orf19.5911, orf19.3278, orf19.403, orf19.2866, orf19.1641, orf19.6321, orf19.1330, orf19.6814, orf19.2475, orf19.4550, orf19.5288, orf19.3888, orf19.769, orf19.6983, orf19.1034, orf19.413, orf19.5917, orf19.6086, orf19.6788, orf19.1796, orf19.1075, orf19.3967, orf19.3325, orf19.5228, orf19.3282, orf19.2251, orf19.4456, orf19.6559, orf19.4580, orf19.1433, orf19.388, orf19.3618, orf19.2674, orf19.5140, orf19.6640, orf19.2337, orf19.1381, orf19.3442, orf19.2261, orf19.6116, orf19.7303, orf19.1326, orf19.5620, orf19.6784, orf19.1275, orf19.6757, orf19.542, orf19.5818, orf19.1802, orf19.734, orf19.5079, orf19.4148, orf19.386, orf19.3053, orf19.1365, orf19.4581, orf19.1743, orf19.3902, orf19.2877, orf19.6937, orf19.7479, orf19.6277, orf19.5524, orf19.320, orf19.5773, orf19.410.3, orf19.6038, orf19.4624, orf19.3940.1, orf19.6731, orf19.6001, orf19.4747, orf19.6082, orf19.4048, orf19.3150, orf19.3781, orf19.2132, orf19.3380, orf19.3969, orf19.36.1, orf19.6305, orf19.1333, orf19.6065, orf19.2529.1, orf19.6562, orf19.6540, orf19.6316, orf19.2023, orf19.3515, orf19.5801, orf19.5612, orf19.6447, orf19.5267, orf19.744, orf19.5078, orf19.6744, orf19.7098, orf19.3038, orf19.6214, orf19.4784, orf19.1723, orf19.7104, orf19.6671, orf19.4617, orf19.5210, orf19.7596, orf19.6673, orf19.4828, orf19.4738, orf19.904, orf19.2107, orf19.5346, orf19.7561, orf19.3066, orf19.7023, orf19.5205, orf19.4444, orf19.7459, orf19.5197, orf19.6656, orf19.4791, orf19.2463, orf19.5519* |
| Up-regulated in acetic and butyric acid | *orf19.3360, orf19.4856, orf19.3358, orf19.147, orf19.3863, orf19.6511, orf19.1850, orf19.1762, orf19.6685, orf19.5856, orf19.2930, orf19.3169, orf19.3103, orf19.5294, orf19.6770, orf19.2068, orf19.5436, orf19.7380, orf19.6591, orf19.3706, orf19.1861, orf19.1335, orf19.5288.1, orf19.2488, orf19.429, orf19.5206, orf19.3304, orf19.600, orf19.1585, orf19.2009, orf19.3831, orf19.3707, orf19.5229, orf19.4286, orf19.5295, orf19.4681, orf19.7160, orf19.2369.1, orf19.5977, orf19.3428, orf19.7343, orf19.3291, orf19.6838, orf19.6710, orf19.1517, orf19.649, orf19.1655.3, orf19.2934, orf19.2545, orf19.541, orf19.3895, orf19.1791, orf19.4607, orf19.7281, orf19.4347, orf19.1954, orf19.2309, orf19.4962, orf19.5925, orf19.3572, orf19.4508, orf19.1466, orf19.3704, orf19.3434, orf19.7392, orf19.742, orf19.5758, orf19.276, orf19.1250, orf19.3110, orf19.1764, orf19.4029, orf19.1288, orf19.2747, orf19.5958, orf19.827, orf19.4219, orf19.5110, orf19.5938, orf19.6247.1, orf19.6830, orf19.2715, orf19.3370, orf19.3330, orf19.1953, orf19.168, orf19.4887, orf19.7300, orf19.6014, orf19.4674.1, orf19.4000, orf19.2314, orf19.970, orf19.5608, orf19.7036, orf19.4363, orf19.2748, orf19.1303, orf19.6902, orf19.6175, orf19.6061, orf19.5672, orf19.275, orf19.1033, orf19.2320, orf19.1403, orf19.6811, orf19.2017, orf19.2852, orf19.2408, orf19.3687, orf19.3609, orf19.5134, orf19.2397.3, orf19.443, orf19.4183, orf19.7215, orf19.6275, orf19.2487, orf19.4863, orf19.6639, orf19.4950, orf19.773, orf19.2847, orf19.6845, orf19.475, orf19.952, orf19.1742, orf19.5605, orf19.7223, orf19.7472, orf19.5552, orf19.2831, orf19.5597, orf19.5759, orf19.4746, orf19.5567, orf19.2711, orf19.2594, orf19.3213, orf19.7481, orf19.2680, orf19.3463, orf19.7332, orf19.3724, orf19.1263, orf19.731, orf19.220, orf19.4896, orf19.227, orf19.2676, orf19.3773, orf19.494, orf19.6141, orf19.2256, orf19.3220, orf19.651, orf19.3929, orf19.685.1, orf19.6234, orf19.4697, orf19.58, orf19.3366.1, orf19.5067, orf19.3564, orf19.5755, orf19.2386, orf19.4757, orf19.3759, orf19.2167, orf19.1029, orf19.4587, orf19.3553, orf19.1833, orf19.5521, orf19.7565, orf19.873.1, orf19.909.1, orf19.5987, orf19.1813, orf19.6072.1, orf19.4965, orf19.7006, orf19.7159, orf19.6322, orf19.6244, orf19.1860, orf19.4593, orf19.665, orf19.1187, orf19.2650, orf19.2607, orf19.2881, orf19.889, orf19.1404, orf19.4494, orf19.3130, orf19.7088, orf19.5238, orf19.2193, orf19.1352, orf19.3298, orf19.5885, orf19.4546, orf19.732, orf19.1499, orf19.1815, orf19.7361, orf19.5000, orf19.2938, orf19.5905, orf19.2706, orf19.5553, orf19.6417, orf19.782, orf19.2489, orf19.4399, orf19.6355, orf19.1267, orf19.1753, orf19.2270, orf19.2654, orf19.1047, orf19.2511.2, orf19.3606, orf19.5550, orf19.1027, orf19.4450.2, orf19.6236, orf19.2117, orf19.4268, orf19.5884, orf19.2385, orf19.7486, orf19.6951, orf19.6249, orf19.7618, orf19.6726, orf19.1642, orf19.7624, orf19.3204, orf19.7664, orf19.7569, orf19.3483, orf19.107, orf19.3756, orf19.1120, orf19.4739, orf19.1140, orf19.2998, orf19.6550, orf19.1028, orf19.4294, orf19.1609, orf19.4451, orf19.6137, orf19.4144, orf19.4468, orf19.3854, orf19.4640, orf19.603, orf19.5730, orf19.872, orf19.198, orf19.3988, orf19.6090, orf19.7298, orf19.2432, orf19.6989, orf19.3978, orf19.4825, orf19.6235, orf19.5792, orf19.5924, orf19.1646, orf19.3300, orf19.6066, orf19.3610, orf19.4188, orf19.5847, orf19.1199, orf19.6308, orf19.718, orf19.1886, orf19.2604, orf19.3170, orf19.5201, orf19.1589, orf19.5213.2, orf19.5500, orf19.2782, orf19.2891, orf19.4966, orf19.3578, orf19.968, orf19.5207, orf19.5912, orf19.5383, orf19.6862, orf19.6418, orf19.3630, orf19.6769, orf19.6987, orf19.446.1, orf19.5217, orf19.1687, orf19.2973, orf19.4210, orf19.3675, orf19.4513, orf19.4759, orf19.6958, orf19.7154, orf19.1375, orf19.5038, orf19.1902, orf19.3865, orf19.3676, orf19.1923, orf19.1440, orf19.3867, orf19.199, orf19.2319, orf19.4365, orf19.286, orf19.4225.1, orf19.4813, orf19.789, orf19.6527, orf19.4760, orf19.2362, orf19.4401, orf19.4101, orf19.868, orf19.3815, orf19.5952, orf19.2972, orf19.2688, orf19.3539, orf19.809, orf19.3288, orf19.2143, orf19.7314, orf19.5353, orf19.4492, orf19.6594, orf19.4940, orf19.5026, orf19.5824, orf19.123, orf19.6480, orf19.1362, orf19.3138, orf19.969, orf19.3778, orf19.661, orf19.5424, orf19.2414, orf19.691, orf19.172, orf19.1091, orf19.6675, orf19.5114.1, orf19.847, orf19.2570, orf19.2917, orf19.1388, orf19.7546, orf19.3941, orf19.1945, orf19.5407, orf19.5566, orf19.5198, orf19.500, orf19.6607, orf19.512, orf19.5232, orf19.2360, orf19.5106, orf19.3665, orf19.5257, orf19.5282, orf19.851, orf19.59, orf19.1109, orf19.3321, orf19.7107, orf19.4328, orf19.133, orf19.932, orf19.6554, orf19.7247, orf19.2599, orf19.4191, orf19.1978, orf19.1082.1, orf19.4015, orf19.1440.2, orf19.3393, orf19.1124.2, orf19.819, orf19.73, orf19.5732, orf19.5507, orf19.5626, orf19.3540, orf19.6298, orf19.1473, orf19.6132, orf19.706, orf19.6035, orf19.2667, orf19.1633, orf19.1617, orf19.4592, orf19.563, orf19.670.2, orf19.3962, orf19.4983, orf19.2250, orf19.1915, orf19.2185, orf19.3088, orf19.2781, orf19.7552, orf19.2306, orf19.5213.1, orf19.1956, orf19.3829, orf19.3827, orf19.3335, orf19.3627, orf19.7599, orf19.2458, orf19.2527, orf19.6898.1, orf19.5785, orf19.341, orf19.3287, orf19.637, orf19.2735, orf19.3794, orf19.638, orf19.2871, orf19.6307, orf19.6555, orf19.2707.1, orf19.1471, orf19.35, orf19.1839, orf19.4143, orf19.5356, orf19.6152, orf19.5894, orf19.1872, orf19.5492, orf19.2090, orf19.1077, orf19.2446, orf19.1575, orf19.1826, orf19.3154, orf19.6660, orf19.2514, orf19.7509.1, orf19.4883, orf19.4775, orf19.3624, orf19.1578, orf19.2435, orf19.6376, orf19.6297, orf19.2712, orf19.760, orf19.4578, orf19.5585, orf19.3470, orf19.339, orf19.501, orf19.5704, orf19.48, orf19.6794, orf19.2726, orf19.2564, orf19.76, orf19.2398, orf19.6886, orf19.4688, orf19.7619, orf19.3223.1, orf19.2287, orf19.4870, orf19.4093, orf19.2717, orf19.5079.1, orf19.6047, orf19.3478, orf19.1770, orf19.4450, orf19.6073, orf19.5991, orf19.7381, orf19.7131, orf19.6659, orf19.655, orf19.5077, orf19.2439, orf19.4077, orf19.1367.1, orf19.2504, orf19.3516, orf19.1719, orf19.4505, orf19.3276, orf19.4479, orf19.1467, orf19.5597.1, orf19.415, orf19.1890, orf19.169, orf19.4490.2, orf19.3290, orf19.4591, orf19.862, orf19.332.1, orf19.3782.2, orf19.4815, orf19.287, orf19.6589, orf19.4012, orf19.1710, orf19.3167, orf19.2829, orf19.7422, orf19.5049, orf19.962, orf19.134, orf19.124, orf19.1549, orf19.1480, orf19.7635, orf19.7114, orf19.5962, orf19.5959, orf19.3547, orf19.1393, orf19.6578, orf19.6766, orf19.5629, orf19.3159, orf19.5299, orf19.94, orf19.6887, orf19.6007, orf19.6928, orf19.6652, orf19.4751.1, orf19.4743, orf19.7215.3, orf19.5070, orf19.3611, orf19.4160, orf19.1438, orf19.4932, orf19.7197, orf19.5064.1, orf19.7062, orf19.6955, orf19.449, orf19.6686, orf19.2810, orf19.873, orf19.2821, orf19.3432, orf19.7077, orf19.4793, orf19.2067, orf19.3527, orf19.2859, orf19.7500, orf19.1896, orf19.6514, orf19.3574, orf19.6585, orf19.5005, orf19.5547, orf19.272, orf19.4758, orf19.6306, orf19.2978, orf19.4396, orf19.3133, orf19.1566, orf19.6828, orf19.4273, orf19.4568, orf19.3015, orf19.4593.1, orf19.318, orf19.3757, orf19.5850, orf19.1150, orf19.2952, orf19.1363, orf19.913.2, orf19.4894, orf19.2091, orf19.804.1, orf19.3171, orf19.3672, orf19.7093, orf19.5419, orf19.7384, orf19.6648, orf19.1682, orf19.2439.1, orf19.813, orf19.823, orf19.6548, orf19.7011, orf19.5893, orf19.1030, orf19.3340, orf19.6837, orf19.4679, orf19.588, orf19.4689, orf19.4826, orf19.2954, orf19.1625, orf19.6531, orf19.3579, orf19.3931, orf19.925, orf19.1701, orf19.5045, orf19.1944, orf19.446.2, orf19.414, orf19.4737, orf19.2575, orf19.2066.1, orf19.5231.2, orf19.711, orf19.7359, orf19.3670, orf19.3507, orf19.4706, orf19.1979, orf19.3934, orf19.6062.3, orf19.3223, orf19.5010, orf19.1486, orf19.4016, orf19.6672, orf19.5565, orf19.4159, orf19.7534, orf19.4600, orf19.93, orf19.3661, orf19.7590, orf19.5660.1, orf19.5491.1, orf19.1873, orf19.1957, orf19.4311, orf19.7434, orf19.4895, orf19.4495, orf19.3710, orf19.558, orf19.2724, orf19.5201.1, orf19.3733, orf19.7056, orf19.2846, orf19.4096, orf19.5216, orf19.3742, orf19.5653, orf19.499, orf19.1397, orf19.1721, orf19.1032, orf19.2785, orf19.2723, orf19.3923, orf19.822, orf19.7566, orf19.4602, orf19.2644, orf19.1430, orf19.4885, orf19.22, orf19.3499, orf19.6854, orf19.930, orf19.6938, orf19.6864, orf19.5805, orf19.4773, orf19.6126, orf19.6408, orf19.6165, orf19.5791, orf19.7411, orf19.1065, orf19.740, orf19.1264, orf19.4017, orf19.1148, orf19.882, orf19.5753, orf19.5447, orf19.5069, orf19.3664, orf19.6385, orf19.1932, orf19.7085, orf19.183, orf19.2624, orf19.4211, orf19.6586, orf19.1117, orf19.3954.1, orf19.4393, orf19.6724, orf19.6311, orf19.4936.1, orf19.5302, orf19.4886, orf19.6486, orf19.4551, orf19.6844, orf19.6484, orf19.7279.1* |
| Down-regulated in acetic and butyric acid | *orf19.689, orf19.2583.2, orf19.2849, orf19.1691, orf19.5674, orf19.5673, orf19.4612, orf19.1442, orf19.5760, orf19.2048, orf19.2602, orf19.4749, orf19.1868, orf19.333, orf19.7106, tE(UUC)1, orf19.4530.1, orf19.4212, orf19.6398, orf19.6679, orf19.510, orf19.1258, orf19.3749, orf19.6998, orf19.2020, orf19.5399, orf19.4690, orf19.4555, orf19.7676, orf19.6637, orf19.539, orf19.2803, orf19.3475, orf19.2584, orf19.5342.2, orf19.3378, orf19.5784, orf19.6570, orf19.3433, orf19.2691, orf19.508, orf19.1097, orf19.903, orf19.4477, orf19.993, orf19.2371, orf19.7283, orf19.5615, orf19.347, orf19.4777, orf19.3575, orf19.4618, orf19.6077, orf19.5573, orf19.3746, orf19.398, orf19.4135, orf19.4943, orf19.5763, orf19.3651, orf19.7196, orf19.4664, orf19.2452, orf19.2114, orf19.2693, orf19.3839, orf19.406, orf19.2738, orf19.1862, orf19.5144, orf19.2745, orf19.2175, orf19.2372, orf19.5616, orf19.1368, orf19.4170, orf19.2335, orf19.434, orf19.1681, orf19.6222.1, orf19.2839, orf19.1325, orf19.2989, orf19.4215, orf19.5549, orf19.1999, orf19.342, orf19.771, orf19.5103, orf19.3915, orf19.5417, orf19.3879, orf19.4836, orf19.3803, orf19.5812, orf19.513, orf19.3122, orf19.6078, orf19.2248, orf19.7310, orf19.7437, orf19.85, orf19.767, orf19.2125, orf19.2047, orf19.84, orf19.7148, orf19.1667.1, orf19.1116, orf19.1995, orf19.176, orf19.5645, orf19.7199, orf19.4665, orf19.7127.1, orf19.3591, orf19.6852.1, orf19.2179, orf19.2474, orf19.6869, orf19.1889, orf19.7323, orf19.1510, orf19.5820, orf19.4044, orf19.2461, orf19.260, orf19.2769, orf19.3916, orf19.4216, orf19.2451, orf19.5025, orf19.2246, orf19.5779, orf19.5841, orf19.3160, orf19.3780, orf19.1341, orf19.2172, orf19.4255, orf19.7580, orf19.4853, orf19.7149, snR33a, orf19.1901* |
| Up-regulated in butyric acid only | *orf19.4767, orf19.3098, orf19.2786, orf19.4801, orf19.5234, orf19.6287, orf19.723, orf19.194, orf19.5767, orf19.4231, orf19.3666, orf19.5021, orf19.1240, orf19.794, orf19.2646, orf19.6530, orf19.1744, orf19.56, orf19.7319, orf19.1519, orf19.721, orf19.4900, orf19.2308, orf19.1026, orf19.7306, orf19.6121, orf19.2619, orf19.6985, orf19.2899, orf19.6831, orf19.5572, orf19.6525, orf19.6920, orf19.440, orf19.3554, orf19.6797, orf19.6347, orf19.2770, orf19.2893, orf19.7282, orf19.4282, orf19.1304, orf19.5865, orf19.4078, orf19.7460, orf19.7398.1, orf19.1756, orf19.7498, orf19.6706, orf19.4199, orf19.4596, orf19.2454, orf19.171, orf19.2073, orf19.5220, orf19.3585, orf19.3327, orf19.4563, orf19.6259, orf19.6435, orf19.834, orf19.866, orf19.3156, orf19.4474, orf19.173, orf19.3727, orf19.6465, orf19.3922, orf19.4318, orf19.3518, orf19.7291, orf19.7173, orf19.1035, orf19.5754, orf19.1395, orf19.1045, orf19.7050, orf19.5864, orf19.1253, orf19.5118, orf19.1214, orf19.3369, orf19.5299.1, orf19.5066, orf19.1793, orf19.7320, orf19.50, orf19.3302, orf19.5976, orf19.6544, orf19.3791, orf19.1771, orf19.5961, orf19.4381, orf19.5159, orf19.2882, orf19.6948, orf19.920, orf19.2789, orf19.2883, orf19.1089, orf19.5640, orf19.54, orf19.1306, orf19.7379, orf19.4662, orf19.1806, orf19.6741, orf19.4304, orf19.2044, orf19.1078, orf19.7288, snR75, orf19.4316, orf19.3097, orf19.634, orf19.4370, orf19.1075.1, orf19.1709, orf19.1847, orf19.6881, orf19.7568, orf19.7440, orf19.5121, orf19.3461, orf19.1985, orf19.6276, orf19.2062, orf19.6561, orf19.3573, orf19.3029, orf19.4438, orf19.1449, orf19.1857, orf19.2192, orf19.692, orf19.5514, orf19.633, orf19.1523, orf19.7156, orf19.6229, orf19.6899, orf19.4673, orf19.2701, orf19.5305, orf19.6254, orf19.1652, orf19.1704, orf19.7520, orf19.4041, orf19.4567, orf19.7231, orf19.1655, orf19.2158, orf19.4526, orf19.2160* |
| Down-regulated in butyric acid only | *orf19.6688, orf19.4055, orf19.258, orf19.2060, orf19.3548.1, orf19.4942, orf19.7151, orf19.1964, orf19.4082, orf19.2356, orf19.5102, orf19.5604, orf19.6191, orf19.3761, orf19.922, orf19.4076, orf19.7668, orf19.5180, orf19.946, orf19.1736, orf19.658, orf19.4169, orf19.7564, orf19.6995, orf19.262, orf19.2885, orf19.7634, orf19.5487, orf19.4105, orf19.2657, orf19.3051, orf19.6323, orf19.7009, orf19.33, orf19.5467, orf19.2241, orf19.5842, orf19.5875, orf19.2796, orf19.2076, orf19.5094, orf19.4099, orf19.4616, orf19.687, orf19.4069, orf19.1445, orf19.6025, orf19.1461, orf19.6177, orf19.1606, orf19.4136, orf19.4132, orf19.4367, orf19.1891, orf19.4945, orf19.2350* |
| Up-regulated in acetic acid and Down-regulated in butyric acid | *orf19.7042* |
| Down-regulated in acetic acid and Up-regulated in butyric acid | *orf19.3684* |

**Table S3. Open-reading frames and non-coding RNAs significantly regulated by inactivation of *MIG1*.** Genes differentially expressed between SC5314 and *mig1Δ* strain in YPD and YPM media. These lists were used to draw the diagram in Figure 2C. Transcript identifiers refer to the *C. albicans* Assembly 21 genome annotation.

| **Condition** | **Genes significantly regulated in the same direction at all time-points** |
| --- | --- |
| Up-regulated in YPD only | *orf19.5307, orf19.4384.1, orf19.4384, orf19.4899, orf19.5308, orf19.3668, orf19.3232, orf19.6084, orf19.4450.1, orf19.999, orf19.4567, orf19.7434, orf19.11, orf19.4914.1, orf19.5447, orf19.6844, orf19.3664, orf19.5000, orf19.7566, orf19.4445, orf19.3311, orf19.3684, orf19.1354, orf19.1353, orf19.3672, orf19.3670, orf19.742, orf19.5805, orf19.6838, orf19.7514, orf19.4527, orf19.1288, orf19.7502, orf19.2020, orf19.638, orf19.5037, orf19.1523, orf19.542, orf19.6888, orf19.4317, orf19.4607, orf19.5288, orf19.6141, orf19.6083, orf19.1352, orf19.4114, orf19.7520, orf19.5005, orf19.2841, orf19.3335, orf19.94, orf19.4894, orf19.4608, orf19.5250, orf19.4898, orf19.1704, orf19.4066, orf19.3829, orf19.6637, orf19.2608, orf19.3974, orf19.6941, orf19.2023, orf19.7481* |
| Down-regulated in YPD only | *orf19.6948, orf19.4477, orf19.5612, orf19.2952, orf19.1417, orf19.409, orf19.4279, orf19.4377, orf19.7610, orf19.431, orf19.5992, orf19.5784, orf19.4215, orf19.3803, orf19.2356, orf19.689, orf19.1926, orf19.4749, orf19.4674.1, orf19.85, orf19.4690, tG(GCC)6, orf19.6864, orf19.2475* |
| Down-regulated in YPD and YPM | *orf19.4318* |
| Up-regulated in YPM only | *snR189c* |

**Table S4. Open-reading frames and non-coding RNAs significantly regulated in response to carbon source in presence of WOAs.** Genes differentially expressed between exposures to acetic or butyric acid in YPD and between exposures to acetic or butyric acid in YPD in YPM media for SC5314 strain. These lists were used to draw the diagram in Figure 2D. Transcript identifiers refer to the *C. albicans* Assembly 21 genome annotation.

| **Condition** | **Genes significantly regulated in the same direction at all time-points** |
| --- | --- |
| Up-regulated in acetic acid only | *orf19.6317, orf19.501, orf19.5906, orf19.5912, orf19.4118, orf19.3335, orf19.6937, orf19.385, orf19.4167, orf19.5061, orf19.1306, orf19.3870, orf19.7218, orf19.6788, orf19.1274, orf19.7554, orf19.7231, orf19.6570, orf19.1255, orf19.111, orf19.6178, orf19.4682, orf19.7481, orf19.6142, orf19.6844, snR69, orf19.4384, orf19.4384.1, orf19.4481, snR57, tP(AGG)1, orf19.5307* |
| Down-regulated in acetic acid only | *orf19.4309, orf19.4907, orf19.675, orf19.789, orf19.3742, orf19.251, orf19.4012, orf19.3104, orf19.3192, orf19.3713, orf19.6817, orf19.1430, orf19.1069, orf19.1800, orf19.2989, orf19.6770, orf19.4056, orf19.4774, orf19.2344, orf19.7680, orf19.2285, orf19.6086, orf19.1887, orf19.5785, orf19.2398, orf19.6888, orf19.5813, orf19.4908, orf19.7150, orf19.1653, orf19.3644, orf19.2030, orf19.6660, orf19.4358, orf19.3301, orf19.7229, orf19.6554, orf19.5877, orf19.2013, orf19.733, orf19.2498, orf19.7381, orf19.4043, orf19.1978, orf19.5379, orf19.3448, orf19.5799, orf19.1623, orf19.1598, orf19.4534, orf19.7210, orf19.3105, orf19.7297, orf19.6124, orf19.7329, orf19.3396, orf19.4370, orf19.2131, orf19.767, orf19.6527, orf19.3706, orf19.4127, orf19.2832, orf19.215, orf19.6658, orf19.2432, orf19.5748, orf19.3815, orf19.1481, orf19.847, orf19.399, orf19.6842, orf19.6557, orf19.2350, orf19.5770, orf19.1945, orf19.5388, orf19.3360, orf19.4883, orf19.4911, orf19.1387, orf19.2607, orf19.4262, orf19.7598, orf19.3737, orf19.6672, orf19.5257, orf19.5605* |
| Up-regulated in acetic and butyric acid | *orf19.7017, orf19.6445, orf19.926, orf19.944, orf19.2885, orf19.3470, orf19.7564, orf19.4041, orf19.5850, orf19.1415, orf19.4739, orf19.7190, orf19.4815, orf19.7011, orf19.2649, orf19.4870, orf19.3015, orf19.3612, orf19.1687, orf19.5921, orf19.6090, orf19.4735, orf19.95, orf19.7384, orf19.4114, orf19.3311, orf19.2192, orf19.4066, orf19.7657, orf19.171, orf19.2809, orf19.94, orf19.403, orf19.6254, orf19.3895, orf19.7219, orf19.1124, orf19.6143, orf19.1701, orf19.2633, orf19.4551, orf19.638, orf19.5037, orf19.2942, orf19.6141, orf19.7434, orf19.7566, orf19.5565, orf19.742, orf19.6083, orf19.4933, orf19.3733, orf19.3974, orf19.4833, orf19.7280, orf19.3931, orf19.4527, orf19.4274, orf19.3668, orf19.7514, orf19.5975* |
| Down-regulated in acetic and butyric acid | *orf19.4980, orf19.6387, orf19.7085, orf19.1979, orf19.6408, orf19.2762, orf19.882, orf19.2877, orf19.1065, orf19.5749, orf19.5674, orf19.6117, orf19.7602, orf19.5612, orf19.391, orf19.3861, orf19.4887, orf19.3575, orf19.7544, orf19.362, orf19.3074, orf19.4054, orf19.339, orf19.4044, orf19.6112, orf19.3302, orf19.1153, orf19.5070, orf19.6191, orf19.1861, orf19.1785, orf19.1235, orf19.340, orf19.4013, orf19.2244, orf19.5700, orf19.6515, orf19.6814, orf19.7654, orf19.1120, orf19.6517, orf19.7473, orf19.4436, orf19.2661, orf19.2050, orf19.4784, orf19.31, orf19.3651, orf19.2308, orf19.3038, orf19.21, orf19.147, orf19.395, orf19.3110, orf19.769, orf19.5620, orf19.797, orf19.5798, orf19.4618, orf19.211, orf19.4800, orf19.851, orf19.3190, orf19.5531, orf19.4951, orf19.2743, orf19.903, orf19.3888, orf19.889, orf19.3355, orf19.6244, orf19.406, orf19.5752, orf19.6121, orf19.6881, orf19.173, orf19.2770.1* |
| Up-regulated in butyric acid only | *orf19.2478, orf19.2000, orf19.929, orf19.2238, orf19.7084, orf19.4963, orf19.708, orf19.4097, orf19.5012, orf19.6679, orf19.1192, orf19.1351, orf19.2601, orf19.7425, orf19.2934, orf19.2385, orf19.3751, orf19.389, orf19.4068, orf19.1960, orf19.318, orf19.7356, orf19.2384, orf19.6146, orf19.6291, orf19.1499, orf19.3239, orf19.6769, orf19.7154, orf19.3986, orf19.2093, orf19.1150, orf19.7546, orf19.6021, orf19.7118, orf19.5367, orf19.2314, orf19.677, orf19.1030, orf19.1047, orf19.727, orf19.6234, orf19.76, orf19.871, orf19.5383, orf19.547, orf19.7068, orf19.2241, orf19.5353, orf19.7148, orf19.2754, orf19.899, orf19.1791, orf19.1923, orf19.6606, orf19.4507, orf19.7300, orf19.6896, orf19.1388, orf19.4401, orf19.1397, orf19.6041, orf19.6298, orf19.6784, orf19.7565, orf19.6754, orf19.124, orf19.5704, orf19.6026, orf19.2602, orf19.4590, orf19.5517, orf19.3252, orf19.6306, orf19.1966, orf19.1957, orf19.2330, orf19.3434, orf19.7550, orf19.5655, orf19.6066, orf19.3676, orf19.5207, orf19.7488, orf19.4159, orf19.4701, orf19.345, orf19.4160, orf19.1447, orf19.1424, orf19.661, orf19.6936, orf19.6402, orf19.1116, orf19.2927, orf19.3331, orf19.3393, orf19.6028, orf19.1839, orf19.7107, orf19.7115, orf19.4769, snR73, orf19.7394, orf19.6610, orf19.3665, orf19.6782, orf19.6916, orf19.7303, orf19.7100, orf19.7371, orf19.6568, orf19.2515, orf19.2119, orf19.3370, orf19.7354, orf19.3221, orf19.2066, orf19.5113, orf19.3547, orf19.6583, orf19.1584, orf19.5056, orf19.4578, orf19.35.1, orf19.1690, orf19.7658, orf19.5905, orf19.1308, orf19.6329, orf19.2611, orf19.4634, orf19.5026, orf19.2859, orf19.7202, orf19.4712, orf19.6169, orf19.29, orf19.7282, orf19.7453, orf19.4760, orf19.5919, orf19.857, orf19.4793, orf19.1642, orf19.6585, orf19.909.1, orf19.4170, orf19.1633, orf19.1362, orf19.7422, orf19.1115, orf19.133, orf19.685, orf19.4002, orf19.3093, orf19.2267, orf19.6229, orf19.7062, orf19.3501, orf19.5102, orf19.2018.1, orf19.7166, orf19.4435, orf19.217, orf19.499, orf19.7494, orf19.813, orf19.7301, orf19.6907, orf19.454, orf19.4647, orf19.5962, orf19.3829, orf19.3468, orf19.7517, orf19.1393, orf19.6192, orf19.1586, orf19.3777, orf19.2158, orf19.2365, orf19.1996, orf19.5614, orf19.1618.1, orf19.6955, orf19.2891, orf19.2830, orf19.6972, orf19.2479, orf19.4856, orf19.3478, orf19.1035, orf19.5959, orf19.3549, orf19.1797, orf19.5876, orf19.6003, orf19.1835, orf19.1830, orf19.663, orf19.5926, orf19.681, orf19.3670, orf19.3780, orf19.1606, orf19.7621, orf19.1708, orf19.2209, orf19.1486, orf19.2919, orf19.6556, orf19.6686, orf19.5732, orf19.6530, orf19.3432.1, orf19.7668, orf19.1091, orf19.22, orf19.2600, orf19.6526, orf19.6813, orf19.1075.1, orf19.6007, orf19.3171, orf19.3207, orf19.3554, orf19.2836, orf19.6724, orf19.5038, orf19.6886, orf19.2883, orf19.4270, orf19.10, orf19.6205, orf19.7200, orf19.2673, orf19.3234, orf19.660, orf19.7106, orf19.5861.1, orf19.2624, orf19.3133, orf19.2417, orf19.1566, orf19.4157, orf19.3195, orf19.5215, orf19.4788, orf19.2724, orf19.7293, orf19.6648, orf19.414, orf19.3732, orf19.2397, orf19.5753, orf19.5326, orf19.3897, orf19.1237, orf19.2739, orf19.5741, orf19.2829, orf19.715, orf19.5050, orf19.2287, orf19.3711, orf19.2216, orf19.4161, orf19.5010, orf19.909, orf19.4128, orf19.668, orf19.1605, orf19.5449, orf19.5454, orf19.4211, orf19.4354, orf19.3089, orf19.4030, orf19.4616, orf19.5805, orf19.6548, orf19.5507, orf19.5536, orf19.4818, orf19.7232, orf19.6443, orf19.6840, orf19.1709, orf19.1673, orf19.6828, orf19.3352, orf19.2062, orf19.4328, orf19.6322, orf19.2978, orf19.5000, orf19.5242, orf19.5166, orf19.687, orf19.4069, orf19.4125, orf19.6627, orf19.3322, orf19.4819, orf19.2681, orf19.6219, orf19.7469, orf19.4367, orf19.3753, orf19.3088, orf19.2738, orf19.5518, orf19.2003, orf19.5500, orf19.5057, orf19.4895, orf19.2059, orf19.3219, orf19.3100, orf19.4162, orf19.5806, orf19.4096, orf19.5216, orf19.1825, orf19.5063, orf19.4504, orf19.6342, orf19.6247, orf19.5934, orf19.6793, orf19.3727, orf19.7197, orf19.7065, orf19.4765, orf19.1901, orf19.4273, orf19.1724, orf19.5270, orf19.6343, orf19.4894, orf19.7281, orf19.6919, orf19.4301, orf19.4945, orf19.4792, orf19.2735, orf19.6873.1, orf19.4592, orf19.2638, orf19.4145, orf19.3538, orf19.781, orf19.4342, orf19.5873, orf19.97, orf19.2752, orf19.5217, orf19.3461, orf19.183, orf19.4568, orf19.7093, orf19.5487, orf19.272, orf19.4105, orf19.262, orf19.576, orf19.658, orf19.3196, orf19.4142, orf19.8278, orf19.4136, orf19.5723, orf19.6022, orf19.5271, orf19.5431, orf19.4477, orf19.4189, orf19.792, orf19.3925, orf19.2125, orf19.5814.1, orf19.6000, orf19.1445, orf19.732, orf19.1720, orf19.1944, orf19.5025, orf19.5020, orf19.7077, orf19.4914.1, orf19.4393, orf19.3885, orf19.3761, orf19.4169, orf19.740, orf19.7307, orf19.4600, orf19.2168, orf19.7112, orf19.4122, orf19.1287, orf19.2657, orf19.558, orf19.6830, orf19.6140, orf19.4212, orf19.2253, orf19.3439, orf19.4802, orf19.2796, orf19.4335, orf19.3627, orf19.4459, orf19.7029, orf19.6311, orf19.2169, orf19.999, orf19.376, orf19.1350, orf19.1985, orf19.7306, orf19.4255, orf19.3684, orf19.1409.2, orf19.6073, orf19.5952, orf19.3710, orf19.695, orf19.6139, orf19.3282, orf19.5779, orf19.7288, orf19.4773, orf19.7111.1, orf19.1089, orf19.2701, orf19.7539, orf19.2810, orf19.1344, orf19.5604, orf19.7634, orf19.7520, orf19.3646, orf19.5636, orf19.6838, orf19.36.1, orf19.4853, orf19.1263, orf19.5611, orf19.2833, orf19.5845, orf19.344, orf19.5634, orf19.1288, orf19.2591, orf19.4807, orf19.2599, orf19.4567, orf19.2509.1, orf19.4591, orf19.4121, orf19.532, orf19.854, orf19.1321, orf19.1704, orf19.4082, orf19.1652, orf19.5635, orf19.6688, orf19.2460, orf19.6518, orf19.3029, orf19.5169, orf19.1449, orf19.4873, orf19.1541, orf19.7156, orf19.265, orf19.853, orf19.5728, orf19.3232, orf19.6420, orf19.1048, orf19.3924, orf19.7379, orf19.7114, orf19.1370, orf19.1655, orf19.7279.1, orf19.2457, orf19.2247, orf19.4886, orf19.6084, orf19.535, orf19.1363, orf19.1932, orf19.2160, orf19.893, orf19.4476, orf19.4505, orf19.3626.1, orf19.4936.1, orf19.3117, orf19.7608, orf19.1239, orf19.7209, orf19.935, orf19.4934* |
| Down-regulated in butyric only | *snR8b, orf19.1691, orf19.508, orf19.5288, orf19.3618, orf19.1258, orf19.1847, orf19.4526, orf19.6816, orf19.1353, orf19.3967, orf19.6540, orf19.4942, orf19.822, orf19.5114.1, orf19.3419, orf19.341, orf19.3997, orf19.6983, orf19.33, orf19.449, orf19.1868, orf19.1067, orf19.1862, orf19.823, orf19.2529.1, orf19.2371, orf19.5079, orf19.1189, orf19.6882, orf19.2839, orf19.5348, orf19.7227, orf19.5437, orf19.4246, orf19.3869, orf19.4900, orf19.2770, orf19.5136, orf19.3325, orf19.386, orf19.7561, orf19.1473, orf19.542, orf19.2173, orf19.5286, orf19.3707, orf19.4998, orf19.3278, orf19.4777, orf19.5305, orf19.2803, orf19.1034, orf19.6745, ITS2, orf19.2107.1, orf19.5514, orf19.1440.2, orf19.2838, orf19.3363, orf19.3433, orf19.6656, orf19.54, orf19.5911, orf19.1253, orf19.1381, orf19.550, orf19.3156, orf19.904, orf19.3441, orf19.4438, orf19.868, orf19.4674.1, orf19.1616, orf19.6559, orf19.6809, orf19.238, orf19.4279, orf19.7612, orf19.4444, orf19.2896, orf19.1314, orf19.3537, orf19.1796, orf19.6640, orf19.5159, orf19.4679, orf19.915, orf19.7319, orf19.1290, orf19.388, orf19.6338, orf19.5961, orf19.489, orf19.6116, orf19.2372, orf19.2261, orf19.6996, orf19.2269, orf19.7225, orf19.691, orf19.543, orf19.3966, orf19.5572, orf19.5992, orf19.6636, orf19.7676, orf19.3411, orf19.2020, orf19.4372, orf19.2296, orf19.633, orf19.2021, orf19.7060, orf19.734, orf19.4738, orf19.5228, orf19.2335, orf19.7284, orf19.2853, orf19.1152, orf19.2691, orf19.6747, orf19.411, orf19.4824, orf19.1183, orf19.7437, orf19.6055, orf19.1032, orf19.5818, orf19.921, orf19.2876, orf19.7444, orf19.5285, orf19.117, orf19.5033, orf19.4699, orf19.6225, orf19.4617, orf19.2190, orf19.4540, orf19.1331, orf19.3643, orf19.3973, orf19.3012, orf19.2023, orf19.5467, orf19.85, orf19.2414, orf19.1745, orf19.7061, orf19.3649, orf19.2336, orf19.220, orf19.2730, orf19.3053, orf19.434, orf19.6077, orf19.6684, orf19.2251, orf19.1517, orf19.2219, orf19.2508, orf19.3922, orf19.7498, orf19.2175, orf19.6671, orf19.6257, orf19.5526, orf19.1867, orf19.1604, orf19.4624, orf19.3810, orf19.2047, orf19.2655, orf19.4837.1, orf19.3699, orf19.7331, orf19.5587, orf19.2132, orf19.3142, orf19.4917, orf19.277, orf19.5275, orf19.5239, orf19.3047, orf19.1504, orf19.7570, orf19.3007.2, orf19.2381, orf19.7567, orf19.4315, orf19.4474, orf19.4307, orf19.229, orf19.6396, orf19.50, orf19.6621, orf19.5103, orf19.1773, orf19.4614, orf19.4995, orf19.4828, orf19.1955, orf19.4171, orf19.1500, orf19.6245, orf19.2334, orf19.1182, orf19.2623, orf19.2618, orf19.1333, orf19.2248, orf19.1460, orf19.719, orf19.2638.1, orf19.7274, orf19.5408, orf19.1110, orf19.557, orf19.6276, orf19.212, orf19.7600, orf19.945, orf19.7499, orf19.1936, orf19.5224, orf19.6349, orf19.867, orf19.1341, orf19.2108, orf19.5722, orf19.1474, orf19.7021, orf19.6898, orf19.6850, orf19.1368, orf19.2948, orf19.4752, orf19.2433, orf19.100, orf19.4235, orf19.897, orf19.7547, orf19.3412, orf19.5857, orf19.2337, orf19.5177, orf19.6447, orf19.2370, orf19.1502, orf19.6690, orf19.2737, orf19.3930, orf19.7638, orf19.4778, orf19.5525, orf19.396, orf19.2670, orf19.4280, orf19.5221, orf19.6243, orf19.5628, orf19.7207, orf19.3281, orf19.448, orf19.7475, orf19.3007, orf19.6555, orf19.775, orf19.5131, orf19.730, orf19.3521, orf19.510, orf19.820, orf19.5680, orf19.2092, orf19.6635, orf19.2710, orf19.2549, orf19.3701, orf19.834, orf19.3811, orf19.6973, orf19.4953, orf19.6905, orf19.923, orf19.2297, orf19.4850, orf19.5003, orf19.524, orf19.7082, orf19.2149, orf19.5617, orf19.7235, orf19.6744, orf19.7251* |
| Down-regulated in acetic and up-regulated in butyric | *orf19.1395, orf19.6586, orf19.1117, orf19.5713, orf19.7091, orf19.7504, orf19.1631, orf19.3526, orf19.7324* |

**Table S5. Identification of genes regulated by *MIG1* inactivation, by WOAs and by carbon source in presence of WOAs.** These lists were used to draw the diagram in Figure 3A. Transcript identifiers refer to the *C. albicans* Assembly 21 genome annotation.

| **Condition** | **Genes significantly regulated in the same direction at all time-points** |
| --- | --- |
| Regulated by *MIG1* alone | *orf19.5307, orf19.4384.1, orf19.4384, orf19.4899, orf19.5308, orf19.3232, orf19.6084, orf19.4450.1, orf19.999, orf19.4567, orf19.11, orf19.4914.1, orf19.4445, orf19.1354, orf19.1353, orf19.7502, orf19.1523, orf19.542, orf19.6888, orf19.4317, orf19.5288, orf19.7520, orf19.2841, orf19.4608, orf19.5250, orf19.4898, orf19.1704, orf19.2608, orf19.6941, orf19.2023, orf19.6948, orf19.1417, orf19.409, orf19.4279, orf19.4377, orf19.7610, orf19.431, orf19.5992, orf19.2356, orf19.1926, tG(GCC)6, orf19.2475, orf19.4318* |
| Regulated by *MIG1* and WOAs | *orf19.5447, orf19.6844, orf19.3664, orf19.5000, orf19.3684, orf19.3672, orf19.3670, orf19.5805, orf19.6838, orf19.1288, orf19.2020, orf19.4607, orf19.1352, orf19.5005, orf19.3335, orf19.4894, orf19.3829, orf19.6637, orf19.7481, orf19.4477, orf19.2952, orf19.5784, orf19.4215, orf19.3803, orf19.689, orf19.4749, orf19.4674.1, orf19.85, orf19.4690, orf19.6864* |
| Regulated by WOAs alone | *orf19.3360, orf19.4856, orf19.3358, orf19.3863, orf19.6511, orf19.1850, orf19.1762, orf19.6685, orf19.5856, orf19.2930, orf19.3169, orf19.3103, orf19.5294, orf19.6770, orf19.2068, orf19.5436, orf19.7380, orf19.6591, orf19.3706, orf19.1335, orf19.5288.1, orf19.2488, orf19.429, orf19.5206, orf19.3304, orf19.600, orf19.1585, orf19.2009, orf19.3831, orf19.3707, orf19.5229, orf19.4286, orf19.5295, orf19.4681, orf19.7160, orf19.2369.1, orf19.5977, orf19.3428, orf19.7343, orf19.3291, orf19.6710, orf19.1517, orf19.649, orf19.1655.3, orf19.2934, orf19.2545, orf19.541, orf19.1791, orf19.7281, orf19.4347, orf19.1954, orf19.2309, orf19.4962, orf19.5925, orf19.3572, orf19.4508, orf19.1466, orf19.3704, orf19.3434, orf19.7392, orf19.5758, orf19.276, orf19.1250, orf19.1764, orf19.4029, orf19.2747, orf19.5958, orf19.827, orf19.4219, orf19.5110, orf19.5938, orf19.6247.1, orf19.6830, orf19.2715, orf19.3370, orf19.3330, orf19.1953, orf19.168, orf19.7300, orf19.6014, orf19.4000, orf19.2314, orf19.970, orf19.5608, orf19.7036, orf19.4363, orf19.2748, orf19.1303, orf19.6902, orf19.6175, orf19.6061, orf19.5672, orf19.275, orf19.1033, orf19.2320, orf19.1403, orf19.6811, orf19.2017, orf19.2852, orf19.2408, orf19.3687, orf19.3609, orf19.5134, orf19.2397.3, orf19.443, orf19.4183, orf19.7215, orf19.6275, orf19.2487, orf19.4863, orf19.6639, orf19.4950, orf19.773, orf19.2847, orf19.6845, orf19.475, orf19.952, orf19.1742, orf19.5605, orf19.7223, orf19.7472, orf19.5552, orf19.2831, orf19.5597, orf19.5759, orf19.4746, orf19.5567, orf19.2711, orf19.2594, orf19.3213, orf19.2680, orf19.3463, orf19.7332, orf19.3724, orf19.1263, orf19.731, orf19.220, orf19.4896, orf19.227, orf19.2676, orf19.3773, orf19.494, orf19.2256, orf19.3220, orf19.651, orf19.3929, orf19.685.1, orf19.6234, orf19.4697, orf19.58, orf19.3366.1, orf19.5067, orf19.3564, orf19.5755, orf19.2386, orf19.4757, orf19.3759, orf19.2167, orf19.1029, orf19.4587, orf19.3553, orf19.1833, orf19.5521, orf19.7565, orf19.873.1, orf19.909.1, orf19.5987, orf19.1813, orf19.6072.1, orf19.4965, orf19.7006, orf19.7159, orf19.6322, orf19.1860, orf19.4593, orf19.665, orf19.1187, orf19.2650, orf19.2607, orf19.2881, orf19.1404, orf19.4494, orf19.3130, orf19.7088, orf19.5238, orf19.2193, orf19.3298, orf19.5885, orf19.4546, orf19.732, orf19.1499, orf19.1815, orf19.7361, orf19.2938, orf19.5905, orf19.2706, orf19.5553, orf19.6417, orf19.782, orf19.2489, orf19.4399, orf19.6355, orf19.1267, orf19.1753, orf19.2270, orf19.2654, orf19.1047, orf19.2511.2, orf19.3606, orf19.5550, orf19.1027, orf19.4450.2, orf19.6236, orf19.2117, orf19.4268, orf19.5884, orf19.2385, orf19.7486, orf19.6951, orf19.6249, orf19.7618, orf19.6726, orf19.1642, orf19.7624, orf19.3204, orf19.7664, orf19.7569, orf19.3483, orf19.107, orf19.3756, orf19.1140, orf19.2998, orf19.6550, orf19.1028, orf19.4294, orf19.1609, orf19.4451, orf19.6137, orf19.4144, orf19.4468, orf19.3854, orf19.4640, orf19.603, orf19.5730, orf19.872, orf19.198, orf19.3988, orf19.7298, orf19.2432, orf19.6989, orf19.3978, orf19.4825, orf19.6235, orf19.5792, orf19.5924, orf19.1646, orf19.3300, orf19.6066, orf19.3610, orf19.4188, orf19.5847, orf19.1199, orf19.6308, orf19.718, orf19.1886, orf19.2604, orf19.3170, orf19.5201, orf19.1589, orf19.5213.2, orf19.5500, orf19.2782, orf19.2891, orf19.4966, orf19.3578, orf19.968, orf19.5207, orf19.5912, orf19.5383, orf19.6862, orf19.6418, orf19.3630, orf19.6769, orf19.6987, orf19.446.1, orf19.5217, orf19.2973, orf19.4210, orf19.3675, orf19.4513, orf19.4759, orf19.6958, orf19.7154, orf19.1375, orf19.5038, orf19.1902, orf19.3865, orf19.3676, orf19.1923, orf19.1440, orf19.3867, orf19.199, orf19.2319, orf19.4365, orf19.286, orf19.4225.1, orf19.4813, orf19.789, orf19.6527, orf19.4760, orf19.2362, orf19.4401, orf19.4101, orf19.868, orf19.3815, orf19.5952, orf19.2972, orf19.2688, orf19.3539, orf19.809, orf19.3288, orf19.2143, orf19.7314, orf19.5353, orf19.4492, orf19.6594, orf19.4940, orf19.5026, orf19.5824, orf19.123, orf19.6480, orf19.1362, orf19.3138, orf19.969, orf19.3778, orf19.661, orf19.5424, orf19.2414, orf19.691, orf19.172, orf19.1091, orf19.6675, orf19.5114.1, orf19.847, orf19.2570, orf19.2917, orf19.1388, orf19.7546, orf19.3941, orf19.1945, orf19.5407, orf19.5566, orf19.5198, orf19.500, orf19.6607, orf19.512, orf19.5232, orf19.2360, orf19.5106, orf19.3665, orf19.5257, orf19.5282, orf19.59, orf19.1109, orf19.3321, orf19.7107, orf19.4328, orf19.133, orf19.932, orf19.6554, orf19.7247, orf19.2599, orf19.4191, orf19.1978, orf19.1082.1, orf19.4015, orf19.1440.2, orf19.3393, orf19.1124.2, orf19.819, orf19.73, orf19.5732, orf19.5507, orf19.5626, orf19.3540, orf19.6298, orf19.1473, orf19.6132, orf19.706, orf19.6035, orf19.2667, orf19.1633, orf19.1617, orf19.4592, orf19.563, orf19.670.2, orf19.3962, orf19.4983, orf19.2250, orf19.1915, orf19.2185, orf19.3088, orf19.2781, orf19.7552, orf19.2306, orf19.5213.1, orf19.1956, orf19.3827, orf19.3627, orf19.7599, orf19.2458, orf19.2527, orf19.6898.1, orf19.5785, orf19.341, orf19.3287, orf19.637, orf19.2735, orf19.3794, orf19.2871, orf19.6307, orf19.6555, orf19.2707.1, orf19.1471, orf19.35, orf19.1839, orf19.4143, orf19.5356, orf19.6152, orf19.5894, orf19.1872, orf19.5492, orf19.2090, orf19.1077, orf19.2446, orf19.1575, orf19.1826, orf19.3154, orf19.6660, orf19.2514, orf19.7509.1, orf19.4883, orf19.4775, orf19.3624, orf19.1578, orf19.2435, orf19.6376, orf19.6297, orf19.2712, orf19.760, orf19.4578, orf19.5585, orf19.501, orf19.5704, orf19.48, orf19.6794, orf19.2726, orf19.2564, orf19.76, orf19.2398, orf19.6886, orf19.4688, orf19.7619, orf19.3223.1, orf19.2287, orf19.4093, orf19.2717, orf19.5079.1, orf19.6047, orf19.3478, orf19.1770, orf19.4450, orf19.6073, orf19.5991, orf19.7381, orf19.7131, orf19.6659, orf19.655, orf19.5077, orf19.2439, orf19.4077, orf19.1367.1, orf19.2504, orf19.3516, orf19.1719, orf19.4505, orf19.3276, orf19.4479, orf19.1467, orf19.5597.1, orf19.415, orf19.1890, orf19.169, orf19.4490.2, orf19.3290, orf19.4591, orf19.862, orf19.332.1, orf19.3782.2, orf19.287, orf19.6589, orf19.4012, orf19.1710, orf19.3167, orf19.2829, orf19.7422, orf19.5049, orf19.962, orf19.134, orf19.124, orf19.1549, orf19.1480, orf19.7635, orf19.7114, orf19.5962, orf19.5959, orf19.3547, orf19.1393, orf19.6578, orf19.6766, orf19.5629, orf19.3159, orf19.5299, orf19.6887, orf19.6007, orf19.6928, orf19.6652, orf19.4751.1, orf19.4743, orf19.7215.3, orf19.3611, orf19.4160, orf19.1438, orf19.4932, orf19.7197, orf19.5064.1, orf19.7062, orf19.6955, orf19.449, orf19.6686, orf19.2810, orf19.873, orf19.2821, orf19.3432, orf19.7077, orf19.4793, orf19.2067, orf19.3527, orf19.2859, orf19.7500, orf19.1896, orf19.6514, orf19.3574, orf19.6585, orf19.5547, orf19.272, orf19.4758, orf19.6306, orf19.2978, orf19.4396, orf19.3133, orf19.1566, orf19.6828, orf19.4273, orf19.4568, orf19.4593.1, orf19.318, orf19.3757, orf19.1150, orf19.1363, orf19.913.2, orf19.2091, orf19.804.1, orf19.3171, orf19.7093, orf19.5419, orf19.6648, orf19.1682, orf19.2439.1, orf19.813, orf19.823, orf19.6548, orf19.5893, orf19.1030, orf19.3340, orf19.6837, orf19.4679, orf19.588, orf19.4689, orf19.4826, orf19.2954, orf19.1625, orf19.6531, orf19.3579, orf19.925, orf19.5045, orf19.1944, orf19.446.2, orf19.414, orf19.4737, orf19.2575, orf19.2066.1, orf19.5231.2, orf19.711, orf19.7359, orf19.3507, orf19.4706, orf19.3934, orf19.6062.3, orf19.3223, orf19.5010, orf19.1486, orf19.4016, orf19.6672, orf19.4159, orf19.7534, orf19.4600, orf19.93, orf19.3661, orf19.7590, orf19.5660.1, orf19.5491.1, orf19.1873, orf19.1957, orf19.4311, orf19.4895, orf19.4495, orf19.3710, orf19.558, orf19.2724, orf19.5201.1, orf19.7056, orf19.2846, orf19.4096, orf19.5216, orf19.3742, orf19.5653, orf19.499, orf19.1397, orf19.1721, orf19.1032, orf19.2785, orf19.2723, orf19.3923, orf19.822, orf19.4602, orf19.2644, orf19.1430, orf19.4885, orf19.22, orf19.3499, orf19.6854, orf19.930, orf19.6938, orf19.4773, orf19.6126, orf19.6165, orf19.5791, orf19.7411, orf19.740, orf19.1264, orf19.4017, orf19.1148, orf19.5753, orf19.5069, orf19.6385, orf19.1932, orf19.183, orf19.2624, orf19.4211, orf19.3954.1, orf19.4393, orf19.6724, orf19.6311, orf19.4936.1, orf19.5302, orf19.4886, orf19.6486, orf19.6484, orf19.7279.1, orf19.2583.2, orf19.2849, orf19.1691, orf19.5673, orf19.4612, orf19.1442, orf19.5760, orf19.2048, orf19.2602, orf19.1868, orf19.333, orf19.7106, tE(UUC)1, orf19.4530.1, orf19.4212, orf19.6398, orf19.6679, orf19.510, orf19.1258, orf19.3749, orf19.6998, orf19.5399, orf19.4555, orf19.7676, orf19.539, orf19.2803, orf19.3475, orf19.2584, orf19.5342.2, orf19.3378, orf19.6570, orf19.3433, orf19.2691, orf19.508, orf19.1097, orf19.993, orf19.2371, orf19.7283, orf19.5615, orf19.347, orf19.4777, orf19.6077, orf19.5573, orf19.3746, orf19.398, orf19.4135, orf19.4943, orf19.5763, orf19.7196, orf19.4664, orf19.2452, orf19.2114, orf19.2693, orf19.3839, orf19.2738, orf19.1862, orf19.5144, orf19.2745, orf19.2175, orf19.2372, orf19.5616, orf19.1368, orf19.4170, orf19.2335, orf19.434, orf19.1681, orf19.6222.1, orf19.2839, orf19.1325, orf19.2989, orf19.5549, orf19.1999, orf19.342, orf19.771, orf19.5103, orf19.3915, orf19.5417, orf19.3879, orf19.4836, orf19.5812, orf19.513, orf19.3122, orf19.6078, orf19.2248, orf19.7310, orf19.7437, orf19.767, orf19.2125, orf19.2047, orf19.84, orf19.7148, orf19.1667.1, orf19.1116, orf19.1995, orf19.176, orf19.5645, orf19.7199, orf19.4665, orf19.7127.1, orf19.3591, orf19.6852.1, orf19.2179, orf19.2474, orf19.6869, orf19.1889, orf19.7323, orf19.1510, orf19.5820, orf19.2461, orf19.260, orf19.2769, orf19.3916, orf19.4216, orf19.2451, orf19.5025, orf19.2246, orf19.5779, orf19.5841, orf19.3160, orf19.3780, orf19.1341, orf19.2172, orf19.4255, orf19.7580, orf19.4853, orf19.7149, snR33a, orf19.1901, orf19.7042* |
| Regulated by WOAs and carbon source | *orf19.147, orf19.1861, orf19.3895, orf19.3110, orf19.4887, orf19.6244, orf19.889, orf19.1120, orf19.4739, orf19.6090, orf19.1687, orf19.851, orf19.3470, orf19.339, orf19.4870, orf19.4815, orf19.5070, orf19.3015, orf19.5850, orf19.7384, orf19.7011, orf19.3931, orf19.1701, orf19.1979, orf19.5565, orf19.3733, orf19.6408, orf19.1065, orf19.882, orf19.7085, orf19.6586, orf19.1117, orf19.4551, orf19.5674, orf19.903, orf19.3575, orf19.4618, orf19.3651, orf19.406, orf19.4044* |
| Regulated by carbon source alone | *orf19.7017, orf19.6445, orf19.926, orf19.944, orf19.2885, orf19.7564, orf19.4041, orf19.1415, orf19.7190, orf19.2649, orf19.3612, orf19.5921, orf19.4735, orf19.95, orf19.2192, orf19.7657, orf19.171, orf19.2809, orf19.403, orf19.6254, orf19.7219, orf19.1124, orf19.6143, orf19.2633, orf19.2942, orf19.4933, orf19.4833, orf19.7280, orf19.4274, orf19.5975, orf19.4980, orf19.6387, orf19.2762, orf19.2877, orf19.5749, orf19.6117, orf19.7602, orf19.391, orf19.3861, orf19.7544, orf19.362, orf19.3074, orf19.4054, orf19.6112, orf19.3302, orf19.1153, orf19.6191, orf19.1785, orf19.1235, orf19.340, orf19.4013, orf19.2244, orf19.5700, orf19.6515, orf19.6814, orf19.7654, orf19.6517, orf19.7473, orf19.4436, orf19.2661, orf19.2050, orf19.4784, orf19.31, orf19.2308, orf19.3038, orf19.21, orf19.395, orf19.769, orf19.5620, orf19.797, orf19.5798, orf19.211, orf19.4800, orf19.3190, orf19.5531, orf19.4951, orf19.2743, orf19.3888, orf19.3355, orf19.5752, orf19.6121, orf19.6881, orf19.173, orf19.2770.1, orf19.1395, orf19.5713, orf19.7091, orf19.7504, orf19.1631, orf19.3526, orf19.7324* |
| Regulated by *MIG1* and carbon source | *orf19.3668, orf19.3311, orf19.7514, orf19.4527, orf19.5037, orf19.6083, orf19.4114, orf19.4066, orf19.3974, orf19.5612* |
| Regulated in all 3 conditions | *orf19.7434, orf19.7566, orf19.742, orf19.638, orf19.6141, orf19.94* |

**Table S6 Primers used in this study.**

| **Primer name** | **Sequence** |
| --- | --- |
| ACT1-RT-F | TGGAAGCTGCTGGTATTGAC |
| ACT1-RT-R | TTCAGCAATACCTGGGAACA |
| HGT16-RT-F | TTTGTATTGTCGACCGGCTA |
| HGT16-RT-R | TGTCACCATTTGCCACTCTT |
| HGT16upF-KpnI | TTTTTTGGTACCGAAGGAACTTCAGGGTTGTAG |
| HGT16upR-ApaI | TTTTTTGGGCCCGTGGCTGATAAGAAAGTGCTC |
| HGT16doF-SacII | TTTTTTCCGCGGCAAAATACCAGAAAGAATACC |
| HGT16doR-SacI | TTTTTTGAGCTCCCACATCTATCTTGCTCTAC |

## File S1

**RPKM values for all genes in all conditions.**

File S1 is available for download at

<http://www.g3journal.org/lookup/suppl/doi:10.1534/g3.117.300238/-/DC1/FileS1.xlsx>

**References**

Cottier, F., A.S. Tan, J. Chen, J. Lum, F. Zolezzi *et al.*, 2015a The transcriptional stress response of Candida albicans to weak organic acids. *G3 (Bethesda)* 5 (4):497-505.

Cottier, F., A.S. Tan, X. Xu, Y. Wang, and N. Pavelka, 2015b MIG1 Regulates Resistance of Candida albicans against the Fungistatic Effect of Weak Organic Acids. *Eukaryotic Cell* 14 (10):1054-1061.
